# Molecular Evolutionary Analysis of Nematode Zona Pellucida (ZP) Modules Reveals Disulfide-Bond Reshuffling and Standalone ZP-C Domains

**DOI:** 10.1093/gbe/evaa095

**Published:** 2020-05-19

**Authors:** Cameron J Weadick

**Affiliations:** Department of Biosciences, University of Exeter, United Kingdom

**Keywords:** gene family evolution, supradomain, domain architecture, cysteine connectivity, nematode cuticle, cuticlin

## Abstract

Zona pellucida (ZP) modules mediate extracellular protein–protein interactions and contribute to important biological processes including syngamy and cellular morphogenesis. Although some biomedically relevant ZP modules are well studied, little is known about the protein family’s broad-scale diversity and evolution. The increasing availability of sequenced genomes from “nonmodel” systems provides a valuable opportunity to address this issue and to use comparative approaches to gain new insights into ZP module biology. Here, through phylogenetic and structural exploration of ZP module diversity across the nematode phylum, I report evidence that speaks to two important aspects of ZP module biology. First, I show that ZP-C domains—which in some modules act as regulators of ZP-N domain-mediated polymerization activity, and which have never before been found in isolation—can indeed be found as standalone domains. These standalone ZP-C domain proteins originated in independent (paralogous) lineages prior to the diversification of extant nematodes, after which they evolved under strong stabilizing selection, suggesting the presence of ZP-N domain-independent functionality. Second, I provide a much-needed phylogenetic perspective on disulfide bond variability, uncovering evidence for both convergent evolution and disulfide-bond reshuffling. This result has implications for our evolutionary understanding and classification of ZP module structural diversity and highlights the usefulness of phylogenetics and diverse sampling for protein structural biology. All told, these findings set the stage for broad-scale (cross-phyla) evolutionary analysis of ZP modules and position *Caenorhabditis elegans* and other nematodes as important experimental systems for exploring the evolution of ZP modules and their constituent domains.

## Introduction

Secreted proteins help cells withstand, react to, and shape external conditions (Agrawal et al. 2010; Naba et al. 2016; Cuesta-Astroz et al. 2017). The extracellular environment can be variable and stressful, and in order to properly function under such challenging conditions, secreted proteins often employ specialized domains that can be repurposed to different ends by being recombined into different protein architectures (Bork et al. 1996; Martin et al. 1998). Obtaining an appreciation of the structural diversity of secreted proteins is key to understanding the many biological processes that extend beyond the cellular membrane. In many cases, however, insights into the biology of secreted protein families derive from restricted and potentially nonrepresentative sets of model proteins (e.g., those linked to particular biomedical conditions, those expressed in already established model systems, and those that can be collected at high levels). Taking a broad, comparative view can uncover important but otherwise overlooked aspects of secreted protein structure and function.

The zona pellucida (ZP) module is a key component of many secreted proteins (Bork and Sander 1992; Plaza et al. 2010; Litscher and Wassarman 2015; Bokhove and Jovine 2018). Named after the mammalian egg coat (from which the first family-members were found), ZP modules mediate extracellular protein–protein interactions. Through these actions, ZP-module-bearing proteins (hereafter referred to simply as “ZPD proteins,” following Litscher and Wassarman [2015]) contribute to a variety of critical cellular and developmental processes, including regulating sperm–egg interactions (Raj et al. 2017), acting as a ligand coreceptor in the Transforming Growth Factor-β (TGFβ)/Bone Morphogenic Protein (BMP) signaling pathway (Lin et al. 2011; Saito et al. 2017), and promoting dendrite elongation during neurogenesis (Heiman and Shaham 2009). Knowledge of ZP module structural biology has increased considerably over the last few years, particularly for ZPD proteins linked to human health (Bokhove and Jovine 2018); mutations in these proteins underlie several human diseases, including hearing loss and renal failure (Verhoeven et al. 1998; Devuyst et al. 2017). However, ZP modules are found throughout the animal kingdom, from mammals to jellyfish (Matveev et al. 2007), and there is still much to learn about their structural and functional diversity, particularly from an evolutionary perspective. For example, their role in gametic interactions implies a link to the evolution of species boundaries (Killingbeck and Swanson 2018), and their role in modulating cell shape suggests a connection to the evolution of morphological diversity (Fernandes et al. 2010).

For most ZPD proteins studied to date, the primary purpose of the ZP module is to polymerize and trigger the formation of fibrous extracellular matrices (Jovine et al. 2002, 2006). Understanding the mechanics of ZP module polymerization is an area of active research, particularly with regard to the roles played by the two domains that comprise a ZP module: ZP-N and ZP-C (named for their respective N- and C-terminal positions) (Bokhove and Jovine 2018). Notably, it has been shown that isolated ZP-N domains can spontaneously polymerize into filaments *in vitro* (Jovine et al. 2006). However, for a complete ZP module to polymerize, it must first be activated. Studies of a few biomedically relevant ZPD proteins such as uromodulin and ZP3 indicate that cleavage of the ZP-C domain’s C-terminal tail is critical to the activation process (Jovine et al. 2004; Schaeffer et al. 2009). First, cleavage severs the connection to the membrane, leading to extracellular release. Second, cleavage disrupts inhibitory interactions within the ZP-C domain that prevent polymerization: Postcleavage dissociation exposes an activating “internal hydrophobic patch” (IHP) that is otherwise buried and suppressed by an “external hydrophobic patch” (EHP) situated within the now-cleaved C-terminal tail (Jovineet al. 2004). These findings led to the notion that the ZP-N domain is the primary agent of protein–protein binding activity, and that the ZP-C domain is a regulator of ZP-N that acts to prevent ill-timed polymerization (Jovineet al. 2006). Under the strictest form of this hypothesis, ZP-C domains serve no independent function and, consequently, would not be expected to be found on their own. Thus far, comparative data support this prediction: ZP-N domains have been found in isolation, whereas ZP-C domains have not (Jovineet al. 2006; Callebaut et al. 2007). However, this model of domain functionality cannot directly apply to ZPD proteins that remain membrane-bound and do not polymerize (e.g., the BMP coreceptor endoglin [Saito et al. 2017]). Moreover, ZP-C domains are capable of folding independently *in vitro* (Lin et al. 2011; Diestel et al. 2013; Bokhove et al. 2016) and they contribute to protein–protein binding interfaces in some ZPD proteins (Hanet al. 2010; Linet al. 2011; Diestelet al. 2013; Okumura et al. 2015). These points combine to suggest that standalone ZP-C domains could in theory prove functional and exist on their own in nature.

ZP modules are characterized by the presence of multiple intradomain disulfide bonds (Bork and Sander 1992). However, the number of cysteine residues found per module varies and this has led to contrary views about how the cysteines connect and whether this variation has any functional effect (Jovine et al. 2005; Yonezawa 2014). ZP modules have often been classified as either Type I or Type II based on the number of cysteines found within the ZP-C domain; these two groups were alleged to have nonnested connectivity patterns, and to differ functionally, with Type II but not Type I modules able to homopolymerize (Boja et al. 2003; Darie et al. 2004; Kanai et al. 2008). However, in light of the solved structures of a few ZP modules and isolated ZP-C domains, it was subsequently argued that there is no reliable distinction between these groups, and that polymerization tendencies are unrelated to cysteine connectivity patterns (Bokhove and Jovine 2018). Rather, Bokhove et al proposed that ZP-C domains typically have a standard set of three disulfide bonds (Cys5–Cys7, Cys6–Cys8, and CysA–CysB), with cysteine variation among ZPD proteins resulting primarily from lineage-specific gains and losses of disulfide pairs.

For example, the ZP module component of the BMP coreceptor endoglin lacks the Cys6–Cys8 and CysA–CysB disulfides found in uromodulin (Saito et al. 2017), whereas additional disulfides associated with lineage-specific insertions have been found in some vertebrate egg-coat proteins (e.g., trout VEα/β and chicken ZP3; Darie et al. 2004; Han et al. 2010). The case of ZP3 is an interesting example, as this family of egg-coat proteins possesses a ZP-C subdomain that introduces four additional cysteine residues that are closely situated both along the sequence and in 3D space. Through protein crystallography of chicken ZP3, Han et al. (2010) showed that disulfide bonds covalently link the ZP-C core to its subdomain. By contrast, the results of earlier mass spectrometric analysis of other vertebrate ZP3 proteins (but not including chicken ZP3) indicated several cases where the subdomain’s cysteines paired only among each other (Boja et al. 2003; Darie et al. 2004; Kanai et al. 2008). If true, this pattern would be consistent with disulfide bond evolution via cysteine swapping, which is believed to be generally rare in nature (Thornton 1981; Rubinstein and Fiser 2008). However, mass spectrometry and crystallography provided contradictory results with regard to cysteine connectivity in mouse ZP2 (Boja et al. 2003; Bokhove et al. 2016), suggesting that an artifactual explanation for the apparent cysteine swapping pattern seen among ZP3 proteins cannot be ruled out. Regardless, the larger-scale comparison of ZP3 with other ZPD proteins provides clear evidence for expanded cysteine connectivity beyond the core set of bonds defined by Bokhove et al. (2016). Finally, ZPD proteins may also vary in the presence/absence of individual cysteines that contribute to intermolecular bonds, such as those involved in endoglin dimerization (Saito et al. 2017). These studies have largely attempted to make sense of variation in ZPD cysteine connectivity through visual inspection of aligned proteins sequences or structures without explicit regard to phylogeny. However, employing a phylogenetic approach may prove useful, say by providing insights into whether particular connectivity patterns represent ancestral versus derived states.

The diversity of ZPD modules found across the animal kingdom derive from a lengthy and complex history of speciation and duplication events that repeatedly provided new opportunities for unexpected structural features to arise. Efforts to test for the presence of isolated ZP-C domains and clear instances of disulfide-bond reshuffling in ZP modules would therefore benefit by taking a broad, phylogeny-informed approach. Recent genome sequencing projects for traditionally “nonmodel” systems provide the data needed for such studies, but thus far this path has not been taken. I set out to address this shortcoming through a molecular evolutionary study of ZP modules in nematodes.

Nematodes are an intriguing group for exploring the evolution and diversity of ZP modules for several reasons. First, the *Caenorhabditis elegans* genome encodes roughly twice as many ZP modules as are found in mammalian and fruit fly genomes, hinting at unexplored structural and functional diversity (Muriel et al. 2003; Cohen et al. 2019). Second, the recent sequencing of dozens of nematode genomes (Coghlan et al. 2019) has provided the raw material needed for a focused exploration of ZP module diversity within one of the animal kingdom’s most species-rich groups. Finally, given the proven suitability of *C. elegans* for genetics research, there is the potential for any insights gained from comparative analysis to be explored experimentally. Indeed, several ZPD proteins have already been characterized in *C. elegans*: These proteins are generally referred to as “cuticlin” or CUT proteins on account of their structural roles in the nematode cuticle (Fujimoto and Kanaya 1973; Sebastiano et al. 1991; Muriel et al. 2003; Sapio et al. 2005; Witte et al. 2015). However, the majority of ZPD proteins in *C. elegans* are simply annotated as CUT-like or CUTL proteins and little is known about their biology. Not surprisingly, even less is known about ZPD protein biology in nematodes beyond *C. elegans*, though it has been suggested that study of cuticlin proteins may aid efforts to pharmacologically attack the cuticles of nematodes that parasitize humans, livestock, and crops (Lewis et al. 1994; Ondrovics et al. 2016).

Through phylogenetic analysis of 1,783 ZP modules from 59 nematode species, I found that the diversity of ZP modules present in *C. elegans* largely reflects the retention of subfamilies that originated and diverged prior to the diversification of modern nematodes. Using this phylogenetic framework, I then uncovered evidence for the evolutionary elaboration of ZP-C cysteine connectivity patterns (involving the modification of an otherwise conserved bond via disulfide-bond reshuffling, and the convergent evolution of novel IHP-stabilizing disulfides) and for the replicated loss of ZP-N domains in independent lineages (providing evidence that standalone ZP-C domains exist in nature, contrary to past predictions and observations). By taking a comparative, evolutionary approach, this work provides new insights into ZP module biology that should benefit efforts to determine ZP module structure–function relationships, in particular the functional role of standalone ZP-C domains. More broadly, this work provides a foundation for future phylogenetic studies aimed at providing an evolutionary classification of ZP modules and domains across the animal kingdom.

## Materials and Methods

I compiled a data set of *C. elegans* ZPD protein sequences and used these to search for homologs in other nematodes. WormBase.org version 259 (Lee et al. 2018) lists 45 genes that encode a “Zona pellucida domain” (i.e., linked to INTERPRO-ID IPR001507, PFSCAN-ID PS51034, PFAM-ID PF00100, and/or SMART-ID SM00241), including 5 *cut* and 29 *cutl* genes. Two of these were dropped from further consideration: *cutl-21* encodes an isolated and highly divergent ZP-N domain (Jovine et al. 2006), whereas *r52.6* seems to have been incorrectly annotated (the PFSCAN motif assignment for R52.6 applies only to its first 40 aa, and BlastP searches did not indicate sequence similarity with other nematode ZPD proteins; results not shown). When multiple isoforms were available, I selected a single variant, choosing whichever introduced the fewest/shortest indels in preliminary alignments of *C. elegans* ZP modules. This approach resulted in a data set of 43 *C. elegans* ZPD proteins (supplementary table 1, Supplementary Material online). As unannotated ZPD proteins would have been missed by the above approach, I then conducted BlastP searches of the *C. elegans* proteome, using, in turn, the ZP modules from each of the 43 annotated ZPD proteins as the query. (Details on ZPD module identification and the BlastP search approach are provided below.) Aside from the already-discounted ZP-N-only protein CUTL-21, these searches did not uncover any additional ZPD proteins (results not shown).

ZPD proteins often include other domains upstream of the ZP module; I isolated *C. elegans* ZP modules using GISMO (ver 2.0), an alignment program that uses a Bayesian approach to extract and align the homologous core regions of sequences that potentially contain nonhomologous flanks and insertions (Neuwald and Altschul 2016). Because GISMO is stochastic, I applied it multiple times (*n* = 5); the positions and lengths of inferred insertions and flanking regions varied among replicates, but all targeted the ZP module, retaining the C-terminal consensus cleavage site (CCS) and excluding upstream domains and the N-terminal trafficking motif. The 43 flank-trimmed (but not insertion-trimmed) sequences from the GISMO run with the longest conserved core (obtained using seed 28270; supplementary file 1, Supplementary Material online) were then used as search queries to detect homologs in 58 additional nematode species through similarity searches of whole-genome predicted protein sets (see supplementary table 2, Supplementary Material online, for data set sources). The similarity searches were conducted using BlastP 2.6.0 (Altschul et al. 1990), with low complexity regions within the query sequences masked using “seg yes -soft_masking true.” After removing subjects best matched by CUTL-21 (the divergent ZP-N-only protein), I filtered the results to retain only those subjects with *E*-values lower than 10^−10^ and total query coverages of at least 75%. Alternative isoforms were filtered to keep only the longest, though this was only possible for species where predicted isoforms were explicitly identified via sequence name suffixes (e.g., “t1” and “t2”).

The final data set of 1,783 full-length ZPD protein sequences (supplementary file 2, Supplementary Material online) was aligned and trimmed using GISMO. One hundred replicate alignments were generated, with key phylogenetic analyses repeated across all replicates; random seeds are provided in supplementary file 3, Supplementary Material online. To avoid subjective judgment from biasing the results, alignments were not manually adjusted in any way. Conservation patterns in the focal alignment (the top-scoring alignment according to log-likelihood ratio [LLR] score; supplementary file 4, Supplementary Material online) were visualized using WebLogo (weblogo.berkeley.edu, last accessed May 23, 2020; Crooks et al. 2004). Throughout the paper, site numbering refers to position in the trimmed focal alignment.

Maximum likelihood (ML) phylogenies were estimated using PhyML via the www.atgc-montpellier.fr/phyml/ (last accessed May 23, 2020) web server, with automated SMS-AIC model selection, a BioNJ starting tree, and SPR topology rearrangements (Guindon et al. 2010; Lefort et al. 2017). Model parameters were fixed at their SMS-AIC estimates during tree search. This process was applied to all 100 replicate alignments; results were combined by generating a majority-rule consensus tree and using branch recovery proportions (BRPs) to quantify branch support. Because BRPs can be downwardly biased by rogue taxa/lineages, BRPs were supplemented by “transfer bootstrap expectations” (TBEs), calculated using BOOSTER (booster.pasteur.fr/new/, last accessed May 23, 2020; Lemoine et al. 2018). The typical methods for estimating branch support on ML trees, namely bootstrapping and aLRT SH-like tests, were not employed as these methods ignore uncertainty in the alignment. *N* = 100 sets of ML branch lengths were estimated for the consensus topology via iqTree 1.6.0 (Nguyen et al. 2015), using, in turn, each replicate alignment and its corresponding SMS-AIC substitution model. Trees were rooted using the Minimal Ancestor Deviation (MAD) method via mad 2.2 (Tria et al. 2017); this approach aims to identify the root position that minimizes deviance in root-to-tip lengths, thereby accounting for heterogeneity in evolutionary rate across the tree (which can mislead the simple midpoint rooting approach). Trees were plotted and analyzed using functions from the ape, phytools, and phangorn R packages (Schliep 2011; Popescu et al. 2012; Revell 2012).

**Fig. 1.**
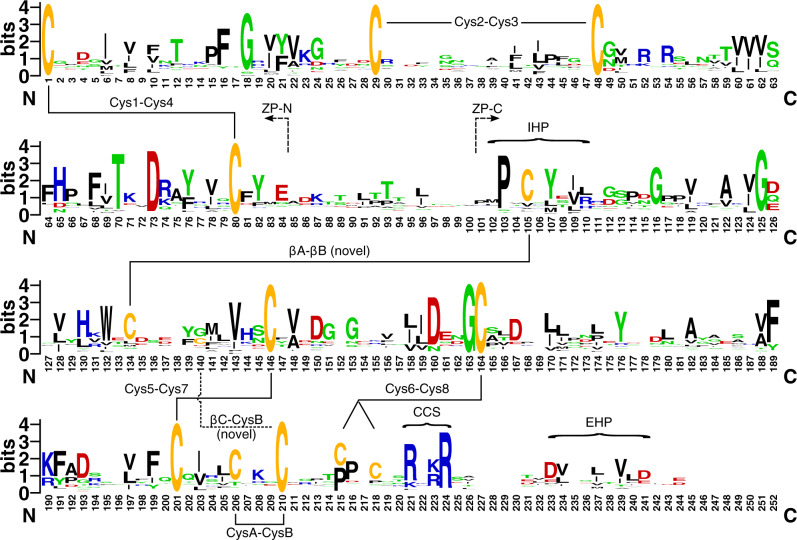
Nematode ZP module amino acid conservation patterns. Residue height indicates its prevalence in the top-scoring alignment. Connections between cysteine residues indicate inferred disulfide linkages; also shown are the approximate boundaries of the ZP-N and ZP-C domains, the internal and external hydrophobic patches (IHP/EHP), and the consensus cleavage site (CCS). Nonhomologous flanks and insertions were trimmed from the sequences as part of the alignment process; the relationship between alignment numbering and untrimmed sequence position for untrimmed *Caenorhabditis elegans* CUT-1 is provided in supplementary figure 1, Supplementary Material online.

Patterns of sequence loss were explored by calculating the amount of missing data within each replicate alignment and mapping these values onto the phylogeny. Gap proportions were estimated separately for the ZP-N and ZP-C domains, with the approximate domain boundaries determined according to cysteine conservation patterns: Using the nomenclature of Bokhove et al. (2016), ZP-N was demarcated using Cys1 and Cys4 (positions 1 and 80; fig. 1), whereas the boundaries of ZP-C were defined using a moderately conserved cysteine in ZP-C’s βA strand along with Cys8 (positions 105 and 218; fig. 1).

Based on the results of the missing-data analysis, three subfamilies were selected for codon model analysis, namely the T01D1.8, F46G11.6, and CUTL-19 subfamilies (named according to their respective *C. elegans* members). In each case, untrimmed protein sequences were realigned using GISMO and the alignment with the top LLR score (out of *n* = 10 replicates) was used to estimate a subfamily-specific phylogeny (via PhyML, as described above) and build a corresponding codon sequence alignment. (The top-scoring alignments were obtained with the following random seeds: T01D1.8 = 25393, F46G11.6 = 21134, and CUTL-19 = 4128.) The codon alignments and trees were used to fit codon substitution models via CodeML from the PAML 4.9a package (Yang 2007). The key parameter for codon models is *ω*, the nonsynonymous (d*N*) to synonymous (d*S*) divergence ratio (=d*N*/d*S*), with values near 0 indicating strong purifying selection and values >1 suggestive of positive selection. I fit three codon models: M8, M8a, and M0. M8 and M8a are nested models that were used to test for site-specific positive selection (*ω* > 1) and to estimate among-site variation in the strength of selective constraint (Swanson et al. 2003); these models were compared via a likelihood ratio test. The simple M0 model assumes that selection is constant across the alignment and was used to obtain overall estimates of the strength of selection (Goldman and Yang 1994) as well as branch-specific estimates of d*S*, which were used to check for saturation. All three models assume that selection is constant across the phylogeny.

Homology models were estimated for *C. elegans* ZP modules using the RaptorX web server (raptorx.uchicago.edu, last accessed May 23, 2020; Kallberg et al. 2012). In most cases, full-length sequences were submitted for analysis: The exceptions were CUTL-19b, T01D1.8b, and F46G11.6 (which are all short, <260 aa long; these sequences were trimmed to remove any predicted N- and C-terminal propeptide flanks), and FBN-1a (which is quite long; only the last 2,500 aa of this 2,799 aa protein were analyzed owing to RaptorX size limits). The 3.2-Å resolution structure for human uromodulin (RCSB PDB code 4wrn; Bokhove et al. 2016) was used as the template for each model; justification for using this template structure is provided in the Results section. When examining the resulting models, I only considered the ZP-N and ZP-C domains, not the up- and downstream regions or the interdomain linker; domain boundaries were determined from each model’s RaptorX structural alignment. Homology models were aligned with one another using DeepAlign:3DCOMB v1.18 (Wang et al. 2011) and then superimposed on the template for visualization and measurement using the “super” function in PyMOL v1.8.6.0 (github.com/schrodinger/pymol-open-source; last accessed May 23, 2020).

C-terminal R/K cleavage sites and N-terminal signaling motifs were predicted for untrimmed sequences via the ProP 1.0 Server (www.cbs.dtu.dk/services/ProP/, last accessed May 23, 2020; Duckert et al. 2004), using a score cutoff of 0.5 and discounting cleavage sites predicted within the signaling peptide. C-terminal GPI-anchors were predicted using PredGPI (gpcr.biocomp.unibo.it/predgpi/pred.htm, last accessed May 23, 2020; Pierleoni et al. 2008), using the “general model” option and a specificity cutoff of 99.0%. Protein domains were predicted using PfamScan (www.ebi.ac.uk/Tools/pfa/pfamscan/, last accessed May 23, 2020; Li et al. 2015) with default search settings.

## Results

### Data Set and Alignment

A data set of 1,783 nematode ZP modules was assembled by BlastP searching the whole-genome predicted protein sets of 58 nematode species for homologs of 43 *C. elegans* ZP modules. The search set included both free-living and parasitic species and covered four of the five major nematode clades defined by Blaxter et al. (1998) (supplementary table 1, Supplementary Material online); by covering such a wide range of species, this approach should hopefully uncover all major nematode ZP module subfamilies regardless of the idiosyncrasies associated with any particular nematode lineage, or the shortcomings associated with any particular genome project. The number of ZP modules per species in the final data set ranged from 15 for *Romanomermis culicivorax* to 58 for *Toxocara canis*, with Clade I nematodes contributing fewer ZP modules to the final data set (median = 21; IQR = 19–22) than Clade III/IV/V species (median = 36; IQR = 28–41).

ZP modules were extracted and aligned using GISMO. Alignment uncertainty is a concern given the short target region and the phylogenetic breadth of the data set. I addressed this by leveraging the stochastic nature of the GISMO alignment procedure, repeating key phylogenetic analyses across 100 replicate alignments. Consistent with expectations for ZP modules, the final GISMO-trimmed alignments were 233–269 aa long, with majority-rule consensus sequences possessing 11–13 cysteines. The percentage of gaps and ambiguous data ranged from 6.7% to 8.6% across alignments. Conservation patterns for the focal alignment (the alignment with the highest LLR score) are shown in figure 1, with the alignment itself available in supplementary file 4, Supplementary Material online. Most alignment sites were highly variable, with several cysteine residues and the ZP-C domain’s R/K-rich CCS being notable exceptions. The relationship between position numbering in the focal alignment and untrimmed sequence position for *C. elegans* CUT-1 is shown in supplementary figure 1, Supplementary Material online.

### Phylogenetics

Evolutionary trees were estimated for the 100 replicate alignments using ML. Alignment variation affected both model selection and the resulting topology. With regard to the substitution model, VT + I + G was favored for 77 alignments, LG + I + G for one, and WAG + I + G+F for the remainder, with the top model receiving an AIC weight of 1.000 in 98/100 cases (supplementary file 3, Supplementary Material online). With regard to the resulting phylogenies, normalized Robinson–Foulds distances ranged from 0.31 to 0.43 between pairs of trees (where 0 corresponds to topologically identical trees and 1 corresponds to completely contradictory trees). However, this method ignores branch lengths; weighting by branch lengths reduced the pairwise Robinson–Foulds distances considerably (range = 0.12–0.23), indicating that many disagreements involved only small-scale differences. The individual trees, including branch lengths and aLRT SH-like partition support values, are provided in supplementary file 5, Supplementary Material online.

**Fig. 2:**
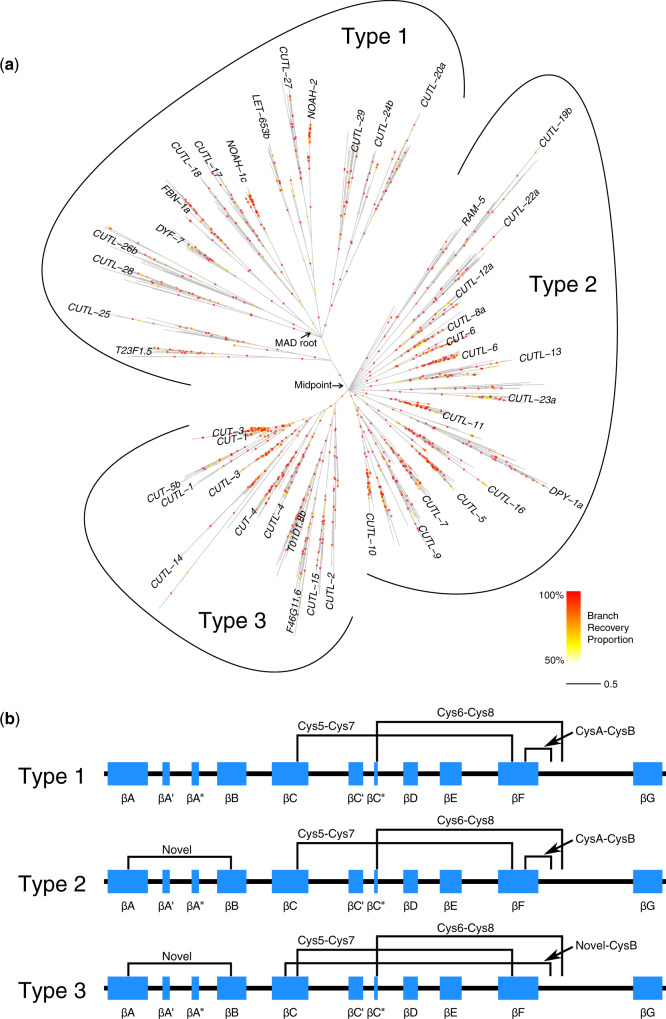
Nematode ZP module phylogeny. (*a*) The majority-rule consensus of ML phylogenies estimated for 100 replicate ZP module alignments. BRPs are shown using colored circles; darker/redder circles indicate greater robustness to alignment variation. Branch lengths, drawn in amino acid substitutions per site (see scale bar), were estimated via ML using the top-scoring alignment. The labeled arrows indicate the Minimal Ancestor Deviation (MAD) root position and the phylogenetic midpoint. Tip names are shown for *Caenorhabditis elegans* ZP modules; for clarity, CUT-1 was moved slightly to avoid overlap with CUT-3. Three major subtrees are noted (Type 1/2/3), the members of which are defined by different ZP-C domain cysteine connectivity patterns. (*b*) Cysteine connectivity patterns for Type 1/2/3 ZP-C domains, inferred based on amino acid conservation patterns and homology modeling of *C. elegans* ZPD proteins. The β-strand secondary structure diagram follows that of the human uromodulin ZP-C domain (Bokhove et al. 2016). The position of the MAD root in (*a*) suggests that the Type 1 connectivity pattern represents the ancestral state.

Rather than focusing on the individual ML trees, I constructed a majority-rule consensus tree (fig. 2*a*), sacrificing resolution for robustness in the face of alignment uncertainty. Doing so reduced the number of internal branches from 1,780 to 1,266 via the formation of 185 polytomies. Most of the retained branches were relatively well supported, with just over half having BRPs of at least 0.95, though 21% had BRPs below 0.70; BRPs are analogous to bootstrap support values but quantify the degree of support for a given branch across replicated estimates of the actual alignment as opposed to bootstrap pseudo-alignments. Phylogenetically unstable branches seem likely for a data set of this size, and these will tend to reduce recovery frequencies for otherwise robust clades. I therefore also estimated TBEs; this approach calculates how frequently each branch is recovered among replicate trees, but it does so in a manner that accounts for rogue branches (Lemoine et al. 2018). Reassuringly, branch support values increased substantially when considering TBE supports, particularly for deeper branches (supplementary figs. 2 and 3, Supplementary Material online). Branch length estimates were largely robust to alignment variation: the majority-rule consensus tree drawn in figure 2*a* shows branch lengths estimated using the focal alignment, but highly similar results were obtained using any of the other 99 replicate alignments (pairwise Pearson’s *r *=* *0.94–0.98).

Visual inspection of the nematode ZP module phylogeny revealed three major groups, which I refer to as Type 1, 2, and 3 ZP modules (fig. 2*a*). These groups are characterized by distinct cysteine conservation patterns that imply alternative ZP-C domain disulfide connectivity patterns (as detailed below via homology modeling) (fig. 2*b* and supplementary fig. 4, Supplementary Material online). The branches that define these three groups are well supported: BRP = 0.90 and TBE = 0.99 for Type 1 versus Type 2/3, and BRP = 0.86 and TBE = 0.99 for Type 2 versus Type 3. The root of the tree was predicted by the MAD method to fall within the Type 1 section of the phylogeny (fig. 2*a* and supplementary fig. 5, Supplementary Material online). Notably, the MAD approach is robust to variation in evolutionary rate among lineages, which appears to be important here (note the long branches within the CUTL-19 and CUTL-14 subfamilies, and the shift between the MAD root and the phylogenetic midpoint that is often used to estimate the root position; fig. 2*a*). This root placement rendered Type 1 modules paraphyletic and therefore suggests that the Type 1 cysteine connectivity pattern is the ancestral state. The Type 2 and 3 modules share a novel pair of ZP-C domain cysteines, and Type 3 modules are further distinguished by the modification of a ZP-C disulfide that remains conserved in Type 1 and 2 modules. Deep relationships within the Type 2 portion of the tree were ambiguous, suggesting a rapid gene family expansion through multiple rounds of duplication and divergence. The consensus tree is equivocal whether Type 2 and 3 modules are sister groups or if Type 2 modules are paraphyletic, with Type 3 modules representing a derived subclade. The latter scenario is supported by the fully resolved tree obtained using the focal alignment, though the short branch lengths and moderate-to-low recovery frequencies made this conclusion uncertain (supplementary fig. 3, Supplementary Material online). Cohen et al. (2019) recently classified *C. elegans* ZP modules into groups based on the number of ZP-C domain cysteine residues present per sequence, and their classification system is broadly congruent with the one provided here (supplementary table 3, Supplementary Material online). However, their approach, which was both nonphylogenetic and *C. elegans*-specific, misclassified a few members that independently lost or gained additional disulfides (detailed below) and did not address which cysteine connectivity pattern is ancestral.

The *C. elegans* ZP modules were, with few exceptions, distributed broadly across the phylogeny, and similar patterns were seen for the other species (fig. 2*a* and supplementary fig. 6, Supplementary Material online). This pattern indicates that the nematode ZP module phylogeny is characterized by over 40 paralogous subfamilies that originated prior to the diversification of modern nematodes, with the members of each subfamily representing clusters of putative orthologs. Indeed, the lengths of the internal branches that connect the various subfamilies are suggestive of ancient origins, perhaps even predating the origin of the nematode phylum. Follow-up studies would therefore do well to sample broadly (i.e., including closely related phyla), as doing so may uncover deep ZP module conservation between invertebrate groups.

Although the tree is largely indicative of stable orthology, occasional lineage-specific gains and losses were also observed. *Caenorhabditis elegans* lacks members of a few subfamilies (i.e., the sister groups to the CUTL-10 and CUTL-23 clades) and the *C. elegans* CUT-1 and CUT-3 modules clearly derive from a recent duplication event. Beyond *C. elegans*, losses appeared particularly common for Clade I nematode ZP modules (supplementary fig. 6, Supplementary Material online). Adaptive gene loss associated with parasitism likely underlies this pattern (Korhonen et al. 2016) but data quality issues also play a role: The short intergenic regions typical of Clade I nematode genomes can cause false fusion events between neighboring genes (Pettitt et al. 2014) and I found that the tandemly arranged *cutl-28* and *dyf-7* genes of Clade I (*Trichinella*) species were fused, resulting in only the DYF-7 sequences ending up in the final data set (results not shown). Putting these few departures aside, the overall pattern is consistent with deep conservation of the ZP module complement across the nematode phylum. Assuming that gene duplication is the primary driver of functional divergence within the ZP module family, these results therefore support efforts to leverage knowledge about cuticular biology in the lab model *C. elegans* for use in treating or preventing parasitic nematode infections.

PfamScan analysis identified a total of 2,310 domains within 1,186 (67%) of the input sequences (supplementary file 2, Supplementary Material online). Most of the predicted matches (91%) were for domains typical of *C. elegans* ZPD proteins, namely the ZP “domain” (Pfam:Zona_pellucida; 36%), two types of PAN domain (Pfam:PAN_1 and PAN_4; 31%), two types of epidermal growth factor (EGF)-like domain (Pfam:EGF_CA and EGF_3; 17%), and the von Willebrand factor Type A (vWFA) domain (Pfam:VWA; 7%). The remaining 9% matched 96 different Pfam entries, with none individually accounting for more than 0.7% of the total; these additional domain predictions were not considered further as nearly half derived from Clade I nematodes (which, as mentioned, have high incidences of artifactually fused genes [Pettitt et al. 2014]). The Pfam:Zona_pellucida entry was only returned for 45% of the sequences, indicating that the domain-prediction approach is prone to false negatives, at least for nematode ZP modules. Mapping the PAN, EGF, and vWFA predictions onto the phylogeny showed that upstream domain predictions within the various subfamilies generally matched expectations, given each clade’s respective *C. elegans* member (supplementary fig. 7, Supplementary Material online). Assuming that domain architecture is conserved within the relevant subfamilies (i.e., that false negatives are more plausible than recurrent domain losses and gains within each subfamily), the majority-rule consensus topology is compatible with single origins for each observed domain architecture. However, the presence of polytomies makes this conclusion tentative for PAN + ZP and vWFA + ZP, and the fully resolved topology estimated using the focal alignment (supplementary fig. 4, Supplementary Material online) actually implies either multiple origins or a single origin followed by multiple losses of the vWFA + ZP arrangement.

### Structural Evolution: Sequence Loss

**Fig. 3:**
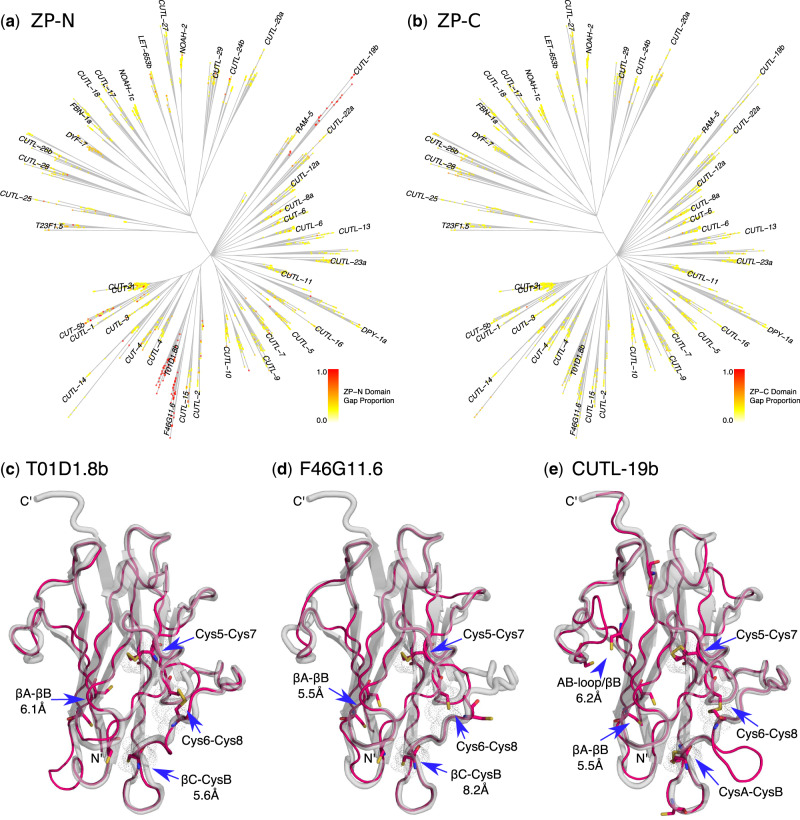
ZP-N domain loss and the structure of standalone ZP-C domain proteins. (*a*, *b*) Domain-specific gap proportions were calculated for each sequence (averaged over the 100 replicate alignments) and mapped onto the phylogeny: (*a*) ZP-N domain and (*b*) ZP-C domain. Gap proportions of 1 (=100%) indicate cases where the entire domain is missing from the core alignment (colored circles on the tips of the phylogeny; see legend). Nearly complete signatures of ZP-N-specific domain loss were observed for the T01D1.8, F46G11.6, and CUTL-19 subfamilies. (*c*–*e*) Homology models for *Caenorhabditis elegans* proteins with standalone ZP-C domains (pink lines) superimposed on the template structure, human uromodulin (gray cartoon): (*c*) T01D1.8b, (*d*) F46G11.6, and (*e*) CUTL-19b. Cysteine residues in the *C. elegans* ZP-C domains are shown in stick format; the three disulfide linkages present in the template (Cys5–Cys7, Cys6–Cys8, and CysA–CysB) are shown as gray dot clouds.

To test for deletions indicative of major structural alterations of the ZP module, I calculated the proportion of missing data for each aligned sequence and mapped these “gap proportions” onto the phylogeny. This was done separately for the ZP-N and ZP-C domains. Three subfamilies—CUTL-19, T01D1.8, and F46G11.6, named for their respective *C. elegans* members—showed pronounced signatures of ZP-N domain sequence loss (fig. 3*a*). Averaged across sequences within the respective subfamilies, ZP-N gap proportion ranged from 83% to 97% for the CUTL-19 subfamily (depending on alignment replicate), 74% to 96% for the T01D1.8 subfamily, and 83% to 99% for the F46G11.6 subfamily. Gap proportions tended to be much lower across the rest of the data set, averaging 6–8% depending on the alignment replicate. Some sequences outside the CUTL-19, T01D1.8, and F46G11.6 subfamilies also showed high gap proportions, but these tended to be local outliers and therefore may simply represent artifactual truncations. For the ZP-C domain, the gap proportion was generally quite low (fig. 3*b*): Averaged across sequences, the gap proportion was 3–4% depending on alignment. Cohen et al. (2019) independently noted the apparent lack of the ZP-N domain in *C. elegans* T01D1.8 and F46G11.6 (no results were reported for CUTL-19) but did not explore the issue further.

The phylogenetic distribution of the standalone ZP-C subfamilies indicates that ZP-N loss occurred prior to the emergence of the major nematode lineages, and that it happened at least twice (fig. 3*a*). The T01D1.8 and F46G11.6 subfamilies are closely related Type 3 modules united by a well-supported branch (BRP = 0.90; TBE = 0.96) and the loss of the ZP-N domain in these subfamilies plausibly represents a single event. The CUTL-19 subfamily, however, is phylogenetically distant, indicating an independent loss of ZP-N within the Type 2 section of the tree. With regard to taxonomic composition, the T01D1.8 subfamily possesses ZP modules from nematodes from all four of the sampled clades (Clades I and III–V), whereas the F46G11.6 and CUTL-19 subfamilies lack sequences from Clade I nematodes (supplementary fig. 6, Supplementary Material online). As Clade I nematodes tend to have considerably fewer ZP modules than other nematodes, this difference presumably reflects two instances of Clade I-specific loss.

Codon model analyses were used to estimate the degree of evolutionary constraint experienced within these three subfamilies. Alignment-wide d*N*/d*S* under the M0 codon model was *ω* = 0.094 for T01D1.8, 0.095 for F46G11.6, and 0.135 for CUTL-19, indicating the action of moderately strong purifying selection acting throughout the history of these subfamilies. Selective constraint was generally strongest within the core regions of the ZP-C domain, especially at sites within predicted β strands (supplementary fig. 8, Supplementary Material online). M8–M8a likelihood ratio tests provided no evidence for site-specific positive selection (*ω* > 1) in any of the subfamilies (supplementary table 4, Supplementary Material online). Under the M0 model, roughly 75% of branches had d*S* < 1 and 98% had d*S* < 3 in each data set, indicating that saturation is unlikely to have strongly affected these analyses.

N-terminal signal peptides were predicted for most members of all three standalone ZP-C domain subfamilies (73% for T01D1.8, 84% for F46G11.6, and 79% for CUTL-19 vs. 66% for the rest of the data set), suggesting that these unusual proteins are still secreted despite the loss of their respective ZP-N domains. However, the three standalone ZP-C subfamilies differed from the norm by generally lacking predicted R/K cleavage sites (30% for T01D1.8, 3% for F46G11.6, and 8% for CUTL-19 vs. 66% for the rest). Examination of the subfamily-specific alignments and *C. elegans* homology models showed that the members of the T01D1.8 and F46G11.6 subfamilies tend to possess short C-terminal tails that terminate before the ZP-C domain’s final β strand, βG, which contains the regulatory EHP motif (fig. 3*c*–*e*). Finally, and unexpectedly, GPI-anchors were predicted for most members of the CUTL-19 subfamily (57%) despite this C-terminal feature being very rare across the rest of the data set (5%, and not found at all in the other two standalone ZP-C subfamilies). Predicted propeptide features for all 1,783 sequences are reported in supplementary file 2, Supplementary Material online.

### Structural Evolution: Cysteine Connectivity

Examination of amino acid variability patterns indicated that some cysteine residues were less strongly conserved than others, suggestive of variation in disulfide binding patterns (fig. 1 and supplementary fig. 4, Supplementary Material online). To explore this further, homology models were generated for the 43 *C. elegans* ZPD proteins using RaptorX (Kallberg et al. 2012). The *C. elegans* ZPD proteins yielded matches to several solved ZP module templates: human uromodulin (RCSB PDB code: 4wrn), chicken ZP3 (3nk3), human endoglin (5hzv), mouse ZP2 ZP-C domain (5bup), and rat betaglycan ZP-C domain (3qw9). I focused only on homology models generated using the human uromodulin template (Bokhove et al. 2016). This was done for three reasons: 1) using a common template facilitated aligning and comparing models generated for different sequences; 2) models built using this template were usually the best option according to RaptorX’s internal ranking system (first place in 34/43 cases and second place in the rest, and always with highly significant model quality *P* values; supplementary table 5, Supplementary Material online); and 3) human uromodulin possesses all three of the putatively typical ZP-C disulfide bonds defined by Bokhove et al. (2016), allowing for evaluation of cysteine connectivity patterns. Homology models and structural alignments are provided in supplementary file 6, Supplementary Material online.

The ZP-N domain was successfully modeled in 39 of 43 cases, the exceptions being the three standalone ZP-C domain proteins plus CUTL-9, which possesses a long insertion within the ZP-N domain’s DE loop that disrupted modeling (supplementary table 5, Supplementary Material online). Structural alignment of the models revealed complete conservation of the two disulfides typical of ZP-N domains, namely the Cys1–Cys4 linkage between the βA and βG strands, and the Cys2–Cys3 linkage between the CD and EE’ loops (supplementary fig. 9, Supplementary Material online). These residues correspond to positions 1, 29, 48, and 80 in the focal alignment, all of which are highly conserved (fig. 1). Examining the positions of other cysteine residues in the *C. elegans* models identified a putative βF–βG disulfide specific to CUTL-5 (supplementary fig. 9, Supplementary Material online). Sequence conservation patterns suggest that this disulfide evolved within the nematode phylum, with the cysteines conserved across Clade III–V orthologs but not Clade I orthologs (results not shown).

The ZP-C domain was successfully modeled in all 43 cases (supplementary table 5 and fig. 10, Supplementary Material online). The models were generally in good agreement with one another, as expected given the use of a common template structure. However, the C-terminal tails often proved difficult to align and model due to the presence of extended FG loops in the *C. elegans* sequences (as can be seen for CUT-1 in supplementary fig. 1, Supplementary Material online; note the long unaligned region immediately prior to the CCS). In some cases, this led to termination of the model prior to the βG strand (the ZP-C domain’s final β strand), even when evidence for it was clearly present in the multiple sequence alignment. In other cases, the βG strand was recovered but connected via a long FG loop that was predicted by RaptorX to have a high propensity for disorder (results not shown). Fortunately, it was still possible to evaluate disulfide binding patterns in most models, as the key cysteine residues are upstream of the poorly modeled region. Doing so revealed clear evidence for large-scale variation in cysteine connectivity among nematode ZP module subfamilies (fig. 2*b*).

According to Bokhove et al. (2016), the typical ZP-C domain has three disulfide bonds: Cys5–Cys7, which connects βC to βF; Cys6–Cys8, which connects βC″ to the FG loop; and CysA–CysB, which connects βF to the FG loop. The Cys5–Cys7 disulfide was recovered in nearly all models (supplementary fig. 10, Supplementary Material online), the only exception being the model for *C. elegans* CUTL-28b. The Cys5 and Cys7 residues are conserved across almost the entire alignment (positions 146 and 201; fig. 1) but both cysteines are absent in the CUTL-28 subfamily (replaced with lysine and alanine, respectively), indicating a subfamily-specific disulfide loss. Cys5–Cys7 loss has also been reported for the *Drosophila* ZPD protein NompA (Bokhove et al. 2016); whether this represents convergent loss or deep conservation awaits cross-phyla phylogenetic analysis, though I note that both NompA and CUTL-28 are predicted to have upstream PAN domains (Fernandes et al. 2010).

The Cys6–Cys8 disulfide was also found to be broadly conserved, though modeling uncertainty makes this conclusion tentative for Type 1 modules. Cys6 mapped to alignment position 164, whereas Cys8 typically mapped to either 215 or 218 (supplementary fig. 4*a* and *b*, Supplementary Material online); a single highly conserved Cys8 alignment column was observed for many alignment replicates (results not shown). A disulfide between Cys6 in the βC″ strand and Cys8 in the FG loop was recovered in all 16 Type 2 modules and in 10 of 12 Type 3 modules (the two exceptions being cases where the unconnected cysteines were placed nearby one another) (supplementary fig. 10*a* and *b*, Supplementary Material online). For Type 1 modules, the Cys6–Cys8 disulfide was recovered (or deemed plausible by proximity) in 7 of 15 models; in the remainder, Cys8 bound or was placed near CysA (supplementary fig. 10*c*, Supplementary Material online). Although this arrangement could indicate a novel connectivity pattern, the fact that it leaves both Cys6 and CysB (the typical partner of CysA) unbound and distant from one another suggests that it is a consequence of inaccurate modeling of the FG loop; notably, these cysteines were all found to be highly conserved across Type 1 modules (supplementary fig. 4*c*, Supplementary Material online). The simplest interpretation is therefore that the Cys6–Cys8 disulfide is conserved in nematode ZPD proteins but is, in some cases, difficult to recover via homology modeling. That said, loss of Cys6–Cys8 has been reported outside of nematodes (e.g., in human endoglin; Saito et al. 2017), indicating that the evolutionary breakdown of the Cys6–Cys8 bond is possible and cannot be conclusively ruled out for all nematode ZPD proteins.

The CysA–CysB disulfide was found to be unexpectedly variable. CysA–CysB, which connects the end of βF strand (position 206) to the beginning of the FG loop (position 210), was recovered in 15 of 16 Type 2 modules (and deemed plausible by proximity in the remaining case) (supplementary fig. 10*b*, Supplementary Material online). Whether this linkage is conserved among Type 1 ZP-C domains is unclear given the FG loop modeling uncertainty described above, though the relevant cysteines are highly conserved (supplementary fig. 4*c*, Supplementary Material online), and the CysA–CysB linkage was recovered for the DYF-7 and LET-653b models (supplementary fig. 10*c*, Supplementary Material online). However, there was a clear loss of the CysA–CysB disulfide in *C. elegans* CUTL-24b; this disulfide has also been lost in some non-nematode ZP proteins (e.g., ZP3; Han et al. 2010) but the example reported here appears to be nematode specific (shared with Clade III–V orthologs, but not with orthologs from Clade I nematodes). The CysA–CysB linkage was also lost in Type 3 modules, albeit in an incomplete manner: Type 3 ZP-C domains lack CysA entirely yet surprisingly retain CysB, which is well positioned to bind a novel cysteine partner in the adjacent βC strand (position 140; median centroid distance of 5.9 Å over the 12 Type 3 models; supplementary figs. 4*a* and 10*a*, Supplementary Material online). These findings strongly suggest that the CysA–CysB disulfide was modified via a partner replacement—partially lost, partially conserved.

**Fig. 4:**
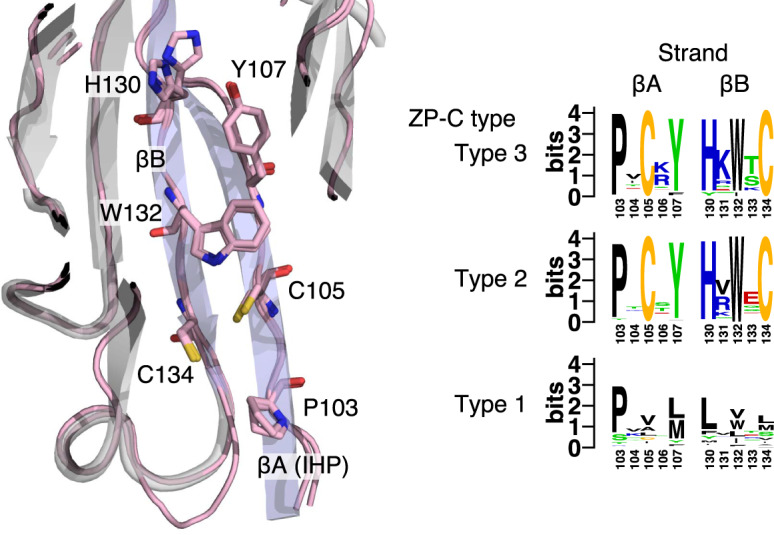
The conserved IHP of Type 2/3 ZP-C domains. Homology models for the ZP-C domains of *Caenorhabditis elegans* CUT-1 (Type 3) and DPY-1a (Type 2) (both shown in pink lines, with key residues shown in stick format) were superimposed on the cartoon structure of the template, human uromodulin (gray cartoon, with the βA and βB strands colored blue). The residues along the inward facing side of βA comprise the IHP; those residues, and the three adjacent residues in βB, are highly conserved in nematode Type 2 and 3 ZP-C domains and suggest a novel disulfide bond. These same sites are variable in Type 1 ZP-C domains. Conservation patterns for the three ZP-C domain types are shown via sequence logos (extracted from supplementary fig. 4, Supplementary Material online).

Beyond the characteristic Cys5–Cys7, Cys6–Cys8, and CysA–CysB disulfides, ZP-C domains sometimes possess additional disulfides, such as the novel Cx–Cy pair found in trout VEα/β egg-coat proteins (Darie et al. 2004) that appears to stabilize a fish-specific expansion of the AB loop, just downstream of the βA-IHP. A few candidates for novel disulfides are apparent in the *C. elegans* ZP-C domain homology models. First, the model for *C. elegans* CUTL-19b included a pair of cysteines that are well placed to link the AB loop and βB (positions 117 and 129; centroid distance = 6.2 Å; fig. 3*e*). These cysteine residues are both conserved across the CUTL-19 subfamily but are not found beyond it, suggesting that stabilizing the AB loop is particularly important in this standalone ZP-C domain subfamily. The second example, which is more broadly distributed (shared across Type 2 and 3 modules), affects the IHP and therefore may be of major functional relevance. Here, cysteines are found at positions 105 (within the IHP motif) and 134 (supplementary fig. 4*a* and *b*, Supplementary Material online). Homology modeling of Type 2 and 3ZP-C domains consistently placed these cysteines near one another: position 105 near the start of βA and position 134 near the end of βB (median centroid distance of 5.6 Å over the 28 Type 2 and 3 models; supplementary fig. 10*a* and *b*, Supplementary Material online). Intriguingly, this putative disulfide forms part of a bipartite motif—one divided between the βA and βB strands—that is highly conserved in Type 2 and 3 ZP modules. Here, three aromatic residues are projected into the same βA–βB interface bridged by the proposed disulfide bond (fig. 4). Finally, a partially overlapping disulfide appears to have evolved within the early history of the FBN-1 subfamily (a Type 1 module). This putative disulfide is defined by cysteines at alignment positions 105 and, uniquely, 203 (centroid distance = 7.3 Å; supplementary fig. 10*c*, Supplementary Material online); both of these cysteines are conserved across the FBN-1 family. A disulfide between these residues would anchor βA not to βB (as seems to be the case for the Type 2 and 3 modules), but to βF. This suggests that similar but not identical disulfide bonds have evolved to stabilize the IHP-containing βA strand in different lineages of the nematode ZP module family.

## Discussion

The ZP module is a supradomain (Vogel et al. 2004)—a combination of structurally independent domains, ZP-N and ZP-C, that function cooperatively and frequently co-occur across a variety of proteins with distinct domain architectures. The co-occurrence between ZP-N and ZP-C is so strong that they were previously considered mere subdomains within a single “ZP domain” (Monne et al. 2008; Han et al. 2010; Bokhove et al. 2016; Wilburn and Swanson 2017), and although isolated ZP-N domains have been found in a variety of proteins, ZP-C domains have only ever been found within complete modules (Jovine et al. 2006). This tight but lopsided distribution is consistent with past studies of ZP structure–function relationships that revealed a role for the ZP-C domain as a regulator of ZP-N activity (Litscher and Wassarman 2015; Bokhove and Jovine 2018). Under the assumption that this regulatory role is the ZP-C domain’s primary function (historically, if not currently in each extant ZPD protein), it makes sense that it would only ever be found immediately downstream of a ZP-N domain. However, studies have uncovered nonregulatory (protein-binding) functions for some ZP-C domains (Han et al. 2010; Lin et al. 2011; Diestel et al. 2013; Okumura et al. 2015; Bokhove et al. 2016), and this raises questions about the apparent lack of standalone ZP-C domains in nature.

I have shown here that standalone ZP-C domains indeed exist—that they can evolve from preexisting ZP modules through ZP-N domain loss. My analysis of nematode ZP modules revealed that standalone ZP-C domain proteins originated at least twice, and that they have been maintained over long timeframes—originating prior to the diversification of the major nematode clades and subsequently evolving under strong stabilizing selection. Despite the loss of the upstream ZP-N domain, these standalone ZP-C proteins generally still possess N-terminal signal peptides, suggesting that they remain secreted proteins. Their C-terminal features, by contrast, are atypical: the members of the T01D1.8 and F46G11.6 subfamilies tend to be truncated, indicating that they may be secreted directly without need for proteolytic separation from the membrane, whereas most members of the CUTL-19 subfamily have predicted GPI-anchor sites (despite this C-terminal feature being rare across the rest of the data set). These findings suggest new dimensions of functionality for ZP-C domains. One possibility is that these standalone ZP-C domains perform a regulatory role, but as free-agent regulators of unlinked ZP-N domains rather than of physically linked upstream domains; such proteins might prove useful for remodeling ZPD protein-based extracellular matrices. Another is that ZP-C domains are multifunctional, having some uncharacterized nonregulatory function. Some ZP-C domains have been shown to contribute to protein–protein binding (Han et al. 2010; Lin et al. 2011; Diestel et al. 2013; Okumura et al. 2015), and it may be that these standalone ZP-C domains do likewise. Either way, the finding that standalone ZP-C domains exist in nature will benefit future experimental efforts to explore the ways in which individual domains contribute to higher-level functioning in ZP module-bearing proteins. The ancient origins for the standalone ZP-C proteins suggest that they might be shared with other phyla, but even if standalone ZP-C domains turn out to be restricted to nematodes alone, the mechanistic insights gleaned from their study will likely prove informative in a general sense.

Of the 43 ZPD proteins encoded by the *C. elegans* genome, less than half have been functionally characterized. Aside from DYF-7, which plays a role in neural dendrite elongation (Heiman and Shaham 2009), all of these are cuticular proteins. Several appear to be cuticlins, that is, noncollageneous structural proteins (Fujimoto and Kanaya 1973; Sebastiano et al. 1991; Muriel et al. 2003; Sapio et al. 2005; Witte et al. 2015), whereas others have been linked to cuticular molting (Frand et al. 2005) or to the development of various cuticular elaborations and invaginations (Yu et al. 2000; Kelley et al. 2015; Gill et al. 2016; Vuong-Brender et al. 2017; Cohen et al. 2019). These cuticular proteins are distributed across the nematode ZP module phylogeny and cover all four of the major domain architectures (ZP, vWFA + ZP, PAN+ZP, and EGF+ZP), suggesting that many of the uncharacterized ZPD proteins, including the standalone ZP-C domain proteins, probably also play a role in the cuticle. Consistent with this hypothesis, transcriptome data from Spencer et al. (2011) and Lee et al. (2018) indicate that T01D1.8 and F46G11.6 are both enriched in the epidermis during early development, but that CUTL-19 is enriched in embryonic and larval motor neurons (suggesting a divergent role, perhaps akin to that of DYF-7). A subsequent study found that T01D1.8 is upregulated in some thermosensitive neurons (Lockhead et al. 2016), hinting at multiple roles for this standalone ZP-C protein. It will be interesting to see, as more nematode ZPD proteins are characterized, whether phylogeny or domain architecture reliably predict functional role, and whether any of these proteins contribute to the egg coat (as ZPD proteins are known to do in vertebrates and at least some invertebrates; Killingbeck and Swanson 2018).

It has been previously shown that artificially isolated ZP-C domains express and fold correctly *in vitro* (Lin et al. 2011; Diestel et al. 2013; Bokhove et al. 2016). The present study provides the first evidence that this experiment has also been performed in nature, with standalone ZP-C domains having evolved from full modules through ZP-N loss. This finding has implications for our understanding of the origin of the ZP module. Two models have been put forth to explain how the original ZP module may have first evolved. The first proposes that the ZP module may have originated via the tandem duplication of a polymerization-capable proto-ZP-N domain, with the C-terminal copy then evolving to form the ZP-C domain (Han et al. 2010). The second hypothesis suggests that ZP modules may have evolved from antibody light chains polypeptides, as both are composed of IG-like domains (Bokhove and Jovine 2018). Finding that standalone ZP-C domains are viable in nature suggests it is possible (though unproven) that such proteins could have independently existed in the deep past, and from this admittedly speculative assumption, two new possibilities arise: 1) the ZP module could have evolved through tandem duplication and divergence of an ancient ZP-C-type domain; and 2) the ZP module could have formed through the union of preexisting but independent ZP-N-type and ZP-C-type domains. Given the lack of recognizable sequence-level homology between ZP-N and ZP-C domains, and between either of these domains and their structurally similar counterparts in antibody light chains, distinguishing among these four models will be difficult. Thorough investigation of the diversity of ZP domains in lineages that connect to the deepest nodes in the animal phylogeny (e.g., non-Bilateria, and possibly even closely related nonanimal groups [Swanson et al. 2011]) will be key to testing these hypotheses.

Identifying highly divergent ZP-C domains will require a good understanding of the domain’s sequence conservation patterns. In practical terms, this amounts to an understanding of cysteine conservation patterns, as most sites beyond these disulfide-forming cysteines are highly variable. Bokhove et al. (2016) argued that cysteine variation in ZP modules largely reflects departures from an otherwise conserved connectivity pattern involving three ZP-C domain disulfides—Cys5–Cys7, Cys6–Cys8, and CysA–CysB—with variation on this theme primarily resulting from occasional losses and gains. This notion is consistent with the general evolutionary patterns observed for disulfide-forming cysteines—that these residues are generally highly conserved, and that they are almost always gained or lost in pairs (Thornton 1981; Rubinstein and Fiser 2008). By contrast, Han et al. (2010) suggested that the novel ZP-C subdomain found in ZP3 egg-coat proteins accommodate alternative cysteine connectivity patterns in different species (though, as mentioned above, methodological issues might explain this apparent pattern; see Introduction). Extracellular proteins with numerous, closely situated cysteines, such as ZPD proteins, seem like promising candidates for identifying unusual instances of disulfide reshuffling.

By combining phylogenetic and structural analyses, I found that disulfide variation among nematode ZP-C domains indeed reflects more than just gains and losses: The CysA–CysB disulfide was modified in Type 3 ZP-C domains, with CysA lost and replaced by a novel binding partner in the adjacent βC strand. The CysB-βC disulfide therefore represents a rare case of disulfide-bond reshuffling (Zhang 2007). Importantly, this modified disulfide is not some recently evolved outlier—it is a feature of multiple ZP module subfamilies (covering 12 *C. elegans* paralogs) that are presumably shared across millions of distantly related nematode species. Given the phylogenetic depth of the branch where this reshuffling event is presumed to have occurred, close inspection of ZPD proteins in other invertebrate phyla might plausibly uncover orthologs that share this connectivity pattern. In light of its ancient origin and subsequent conservation across multiple subfamilies, it seems safe to conclude that stabilizing selection has acted to maintain the modified disulfide bond over time. However, it is not obvious whether the modified disulfide’s initial origin was adaptive, and whether its evolution resulted in some novel function. For example, the evolution of an extra cysteine residue in the vicinity of CysB could have rendered CysA redundant, allowing for its exchange by drift. Another possibility is that the novel CysB-partner evolved to compensate for the loss of CysA; here, the novel disulfide would be adaptive only in the sense that it corrected some transient maladaptation, with no net change in overall function. Regardless, this finding speaks to the challenges of categorizing proteins using sequence conservation patterns without a robust phylogenetic framework, and to the importance of utilizing new data to update expectations about protein biology.

In contrast, there are several reasons to suspect that the entirely novel disulfide inferred between the βA and βB strands of Type 2 and 3 ZP-C domains is adaptive. First, it occurs in a region of known functional importance: the βA-IHP. Stabilizing the IHP through a disulfide bond could help maintain the tertiary structure of the ZP-C domain upon protein maturation and activation, during which the cleaved C-terminal tail’s βG-EHP dissociates from the IHP (Jovine et al. 2004; Schaeffer et al. 2009). Second, it is notable that an IHP-stabilizing disulfide evolved independently within the FBN-1 subfamily (a Type 1 module). Convergent evolution is considered one of the strongest forms of observational evidence for adaptation and it seems unlikely that IHP-stabilizing disulfides would evolve repeatedly without providing some benefit to ZP-C domain structure or function. And finally, there is a clear pattern of coevolution at several nearby sites alongside the same face of the βA and βB strands. These sites are largely fixed for aromatic residues in Type 2 and 3 modules. Fixing aromatic residues along the βA and βB strands may help to stabilize the βA–βB disulfide, act to slow EHP dissociation, or specify a critical interprotein binding surface that is only exposed after EHP release (Bhattacharyya et al. 2004; Moreira et al. 2013). Interestingly, it was recently demonstrated that disulfide bonds act as an evolutionary buffer, increasing tolerance for amino acid substitutions that would have ordinarily been structurally disruptive (Feyertag and Alvarez-Ponce 2017); the fixation of several aromatic residues around the novel βA–βB disulfide provides a clear counterexample to this claim. Determining the functional and evolutionary consequences of these convergently evolved disulfide bonds has the potential to provide important insights into the how IHP–EHP interactions affect ZP module activation.

The present study serves as the largest comparative investigation of ZP module evolutionary diversity conducted to date. By combining the newly estimated nematode ZP module phylogeny with homology modeling of *C. elegans* ZPD proteins, I uncovered evidence for 1) the parallel loss of the ZP-N domain in at least two lineages, resulting in the unexpected discovery of standalone ZP-C domains; 2) the modification of a highly conserved ZP-C domain disulfide via a rare example of cysteine replacement; and 3) the convergent gain of stabilizing disulfide bonds in the ZP-C domain’s regulatory IHP motif. As a purely *in silico* study, it is of course critical that the unusual structural features documented here be confirmed experimentally. Even still, these findings have important implications for our understanding of ZP module structure and function. Moreover, the present study presents a valuable phylogenetic framework for the developmental genetic study of ZPD proteins in nematodes, including the powerful lab model *C. elegans*. Finally, this work sets the stage for future investigation of ZPD protein diversity in the broad sense. Here, the obvious next step will be to bridge the phylogenetic gap between nematodes, other invertebrates and, ultimately, vertebrates.

## Supplementary Material

evaa095_Supplementary_Data
