# Supplementary material for: Molecular Evolutionary Analysis of Nematode Zona Pellucida (ZP) Modules Reveals Disulfide-Bond Reshuffling and Standalone ZP-C Domains
Source: Genome Biol Evol. 2020 May 19;12(8):1240–55. doi: 10.1093/gbe/evaa095 (PMC7456536; doi:10.1093/gbe/evaa095)
Supplement: evaa095_Supplementary_Data [file evaa095_supplementary_data.pdf]

Supplementary Material for:

Molecular evolutionary analysis of nematode Zona Pellucida (ZP) modules reveals disulfide-bond reshuffling and standalone ZP-C domains

Supplementary Files

- Available via the Dryad Digital Repository
  - https://datadryad.org/stash/dataset/doi:10.5061/dryad.q2bvq83g9
  - Weadick, CJ (2020). Data from: Molecular evolutionary analysis of nematode Zona Pellucida (ZP) modules reveals disulfide-bond reshuffling and standalone ZP-C domains, Dryad, Dataset, https://doi.org/10.5061/dryad.q2bvq83g9

1. Preliminary *C. elegans* ZP module alignment
2. Sequence data table
3. Alignment data table
4. Nematode ZP module focal alignment
5. ZP module phylogenies
6. *C. elegans* ZP homology models and structural alignments

Supplementary Tables

1. *C. elegans* ZPD proteins
2. Data set sources
3. Phylogeny-based versus sequence-based classification of nematode ZP modules
4. Codon model analysis of standalone ZP-C domain subfamilies
5. RaptorX homology modelling results

Supplementary Figures

1. Positional map between trimmed and untrimmed versions of *C. elegans* CUT-1
2. Branch support metrics for the majority rule consensus tree
3. Branch support metrics for the focal alignment's maximum likelihood tree
4. Variable amino acid conservation patterns among nematode ZP modules
5. Minimal Ancestor Deviation (MAD) rooting of the nematode ZP module tree
6. Phylogenetic distribution of ZP modules across the major nematode clades
7. Phylogenetic distribution of nematode ZPD protein domain architecture
8. Selective constraint profiles for standalone ZP-C proteins
9. Conserved disulfide connectivity patterns in nematode ZP-N domains
10. Variable disulfide connectivity patterns in nematode ZP-C domains

Supplementary Files

Supplementary File 1: Preliminary *C. elegans* ZP module alignment. A preliminary alignment of *C. elegans* ZPD proteins was generated using GISMO (Neuwald & Altschul 2016 *PLoS Comp Biol* vol. 12) in order to isolate the ZP modules. The top-scoring alignment (highest LLR score) out of five replicates is provided here in GISMO .cma format (obtained using random seed 28270). For each entry, the sequence data within the two sets of parentheses indicate the upstream and downstream flanks. For the data between these two regions, uppercase letters and dashes indicate the homologous core, while lowercase letters indicate insertions within the core.

Supplementary File 2: Sequence data table. A .csv format table providing relevant data for each of the 1783 nematode ZPD protein sequences included in the final data set. Columns indicate: (1) sequence name; (2) species; (3) taxonomic clade (Blaxter et al 1998 *Nature* vol. 392); (4) predicted signal peptide (Y/N); (5) predicted R/K cleavage sites, excluding any predicted within the signal peptide (Y/N); (6) predicted GPI anchor (Y/N); (7) ZP-N domain gap proportion (averaged across alignment replicates); (8) ZP-C domain gap proportion (averaged across alignment replicates); (9) Predicted Pfam domains (significant hits only).

Supplementary File 3: Alignment data table. A .csv format table providing relevant data for each of the 100 replicate alignments estimated for the final data set of 1783 nematode ZP modules. Columns indicate: (1) replicate number; (2) GISMO random seed; (3) length of trimmed alignment (in amino acids); (4) LLR for trimmed alignment; (5) selected substitution model; (6) AIC weight for substitution model; (7) PhyML random seed; (8) ML phylogeny log-likelihood.

Supplementary File 4: Nematode ZP module focal alignment. The top-scoring alignment (i.e., the replicate with the highest LLR score; replicate #38), shown in GISMO .cma format (Neuwald & Altschul 2016 *PLoS Comp Biol* vol. 12). For each entry, the sequence data within the two sets of parentheses indicate the upstream and downstream flanks. For the data between these two regions, uppercase letters and dashes indicate the conserved core, while lowercase letters indicate insertions within the core.

Supplementary File 5: ZP module phylogenies. Trees 1 and 2: majority-rule consensus topology with branch lengths estimated using the alignment with the highest LLR score; support values on tree 1 indicate Branch Recovery Proportions (BRPs), while those on tree 2 indicate Transfer Bootstrap Expectations (TBEs). Trees 3 and 4: the ML topology and branch lengths estimated using the alignment with the highest LLR score (with BRP and TBE support values, respectively). Trees 5–104: ML topologies and branch lengths estimated using the 100 replicate alignments, with aLRT SH-like support values. Alignment replicate #38 received the highest LLR score (=tree #42). All trees are provided in Newick (.nwk) format.

Supplementary File 6: *C. elegans* ZP homology models and structural alignments. Compressed archive (.tar.gz format) containing ZP homology models (.pdb format) and model-template sequence alignments (.fasta format) for *C. elegans* ZPD proteins, using human uromodulin (PDB 4wrnA) as the template. Homology models were generated using RaptorX (Kallberg et al 2012 *Nat Protoc* vol 7). Also included are structural alignments of the models (.pdb format), focussed either on the ZP-N domain (prefix = "zpn1_") or on the ZP-C domain (prefix = "zpc1_"), along with associated input and output files.

Supplementary Tables

Supplementary Table 1: *C. elegans* ZPD proteins.

Protein	Number of Isoforms	Length (aa)[a]	Upstream Domains[b]	Signal Peptide[c]	R/K Cleavage Sites[c,d]	GPI Anchor[e]
**CUT-1**	1	424	—	Y	Y	N
**CUT-3**	1	389	—	Y	N	N
**CUT-4**	1	507	—	Y	N	N
**CUT-5b**	2	379	—	Y	Y	N
**CUT-6**	1	572	vWFA	Y	N	N
**CUTL-1**	1	399	—	N	Y	N
**CUTL-2**	1	382	—	Y	Y	N
**CUTL-3**	1	405	—	Y	Y	N
**CUTL-4**	1	469	—	Y	N	N
**CUTL-5**	1	437	—	Y	Y	N
**CUTL-6**	1	374	—	Y	Y	N
**CUTL-7**	1	585	—	Y	N	N
**CUTL-8a**	2	625	—	Y	N	N
**CUTL-9**	1	562	—	Y	Y	N
**CUTL-10**	1	403	—	Y	Y	N
**CUTL-11**	1	384	—	Y	N	N
**CUTL-12a**	3	569	—	Y	Y	N
**CUTL-13**	1	445	—	Y	Y	N
**CUTL-14**	1	270	—	Y	N	N
**CUTL-15**	1	385	—	Y	Y	N
**CUTL-16**	1	488	—	Y	N	N
**CUTL-17**	1	912	PAN (x3)	Y	Y	N
**CUTL-18**	1	801	PAN (x4)	Y	Y	N
**CUTL-19b**	2	237	—	Y	N	Y
**CUTL-20a**	2	360	—	Y	Y	Y
**CUTL-22a**	3	445	—	Y	N	N
**CUTL-23a**	2	773	vWFA	Y	Y	N
**CUTL-24b**	2	601	—	Y	Y	N
**CUTL-25**	1	385	—	Y	Y	N
**CUTL-26b**	2	502	—	Y	N	N
**CUTL-27**	1	969	PAN (x5)	Y	Y	N
**CUTL-28**	1	696	PAN (x3)	Y	N	N

**CUTL-29**	1	390	—	Y	Y	N
**DPY-1a**	2	1387	vWFA	Y	N	N
**DYF-7**	1	446	—	Y	Y	N
**F46G11.6**	1	160	—	Y	N	N
**FBN-1a**	10	2779	EGF-like (x31)	Y	Y	N
**LET-653b**	3	653	PAN (x2)	Y	N	N
**NOAH-1c**	3	1052	PAN (x6)	Y	Y	N
**NOAH-2**	1	741	PAN (x4)	Y	Y	N
**RAM-5**	1	711	—	Y	N	N
**T01D1.8b**	2	189	—	Y	N	N
**T23F1.5**	1	1262	—	Y	Y	Y

Notes

a - Length of the selected isoform.
b - According to the protein's entry on http://www.WormBase.org.
c - Predicted using Prop 1.0 (http://www.cbs.dtu.dk/services/ProP/).
d - Ignoring any cleavage sites predicted within the signal peptide.
e - Predicted using PredGPI (http://gpcr.biocomp.unibo.it/predgpi/).

Supplementary Table 2. Data set sources. Whole-genome predicted protein sequence data sets (and corresponding coding sequence data sets) were obtained for 59 nematode species from the specified data sources (and corresponding URLs). 'Clade' refers to the species' phylogenetic grouping according to the 5-clade framework of Blaxter et al. (1998 *Nature* vol. 392). '# Proteins' refers to the total number of entries in the predicted protein set; '# modules' refers to the number of sequences included in the final ZP module data set after filtering out annotated isoforms.

Species	Clade	Data Source	URL	Identifier	Assembly Name	# Proteins	# Modules
Ancylostoma ceylanicum	V	parasite.wormbase.org	ftp://ftp.ebi.ac.uk/pub/databases/wormbase/parasite/releases/WBPS9	PRJNA231479	Acey_2013.11.30.genDNA	65583	38
Brugia malayi	III	wormbase.org	ftp://ftp.wormbase.org/pub/wormbase/releases/WS259	PRJNA10729	Bmal-4.0	13436	36
Bursaphelenchus xylophilus	IV	parasite.wormbase.org	ftp://ftp.ebi.ac.uk/pub/databases/wormbase/parasite/releases/WBPS9	PRJEA64437	ASM23113v1_submitted	17704	40
Caenorhabditis angaria	V	wormbase.org	ftp://ftp.wormbase.org/pub/wormbase/releases/WS259	PRJNA51225	ps1010rel8	33934	23
Caenorhabditis brenneri	V	wormbase.org	ftp://ftp.wormbase.org/pub/wormbase/releases/WS259	PRJNA20035	C_brenneri-6.0.1b	30672	41
Caenorhabditis briggsae	V	wormbase.org	ftp://ftp.wormbase.org/pub/wormbase/releases/WS259	PRJNA10731	CB4	25387	54
Caenorhabditis elegans	V	wormbase.org	ftp://ftp.wormbase.org/pub/wormbase/releases/WS259	PRJNA13758	WBcel235	28197	43
Caenorhabditis japonica	V	wormbase.org	ftp://ftp.wormbase.org/pub/wormbase/releases/WS259	PRJNA12591	C_japonica-7.0.1	35976	36
Caenorhabditis monodelphis	V	caenorhabditis.org	http://download.caenorhabditis.org/v1/sequence/	JU1667_v1	v1	21645	45
Caenorhabditis remanei	V	wormbase.org	ftp://ftp.wormbase.org/pub/wormbase/releases/WS259	PRJNA53967	C_remanei-15.0.1	31450	44
Caenorhabditis sinica	V	wormbase.org	ftp://ftp.wormbase.org/pub/wormbase/releases/WS259	PRJNA194557	Caenorhabditis_sp_5-JU800-1.0	46280	40
Caenorhabditis tropicalis	V	wormbase.org	ftp://ftp.wormbase.org/pub/wormbase/releases/WS259	PRJNA53597	Caenorhabditis_sp11_JU1373-3.0.1	27721	36
Dictyocaulus viviparus	V	parasite.wormbase.org	ftp://ftp.ebi.ac.uk/pub/databases/wormbase/parasite/releases/WBPS9	PRJNA72587	D_viviparus_9.2.1.ec.pg	13514	24
Dirofilaria immitis	III	parasite.wormbase.org	ftp://ftp.ebi.ac.uk/pub/databases/wormbase/parasite/releases/WBPS9	PRJEB1797	nDi.2.2	12857	30

Ditylenchus destructor	IV	parasite.wormbase.org	ftp://ftp.ebi.ac.uk/pub/databases/wormbase/parasite/releases/WBPS9	PRJNA312427	ASM157970v1	13938	28
Globodera pallida	IV	parasite.wormbase.org	ftp://ftp.ebi.ac.uk/pub/databases/wormbase/parasite/releases/WBPS9	PRJEB123	GPAL001	16403	18
Globodera rostochiensis	IV	parasite.wormbase.org	ftp://ftp.ebi.ac.uk/pub/databases/wormbase/parasite/releases/WBPS9	PRJEB13504	nGr	14309	27
Haemonchus contortus	V	parasite.wormbase.org	ftp://ftp.ebi.ac.uk/pub/databases/wormbase/parasite/releases/WBPS9	PRJEB506	Haemonchus_contortus_MHco3-2.0	24747	44
Heterorhabditis bacteriophora	V	parasite.wormbase.org	ftp://ftp.ebi.ac.uk/pub/databases/wormbase/parasite/releases/WBPS9	PRJNA13977	Heterorhabditis_bacteriophora-7.0	20964	18
Loa loa	III	parasite.wormbase.org	ftp://ftp.ebi.ac.uk/pub/databases/wormbase/parasite/releases/WBPS9	PRJNA60051	Loa_loa_V3	15445	29
Meloidogyne hapla	IV	parasite.wormbase.org	ftp://ftp.ebi.ac.uk/pub/databases/wormbase/parasite/releases/WBPS9	PRJNA29083	Freeze_1	14420	24
Meloidogyne incognita	IV	parasite.wormbase.org	ftp://ftp.ebi.ac.uk/pub/databases/wormbase/parasite/releases/WBPS9	PRJEA28837	ASM18041v1a	20365	30
Necator americanus	V	parasite.wormbase.org	ftp://ftp.ebi.ac.uk/pub/databases/wormbase/parasite/releases/WBPS9	PRJNA72135	N_americanus_v1	19153	18
Onchocerca volvulus	III	wormbase.org	ftp://ftp.wormbase.org/pub/wormbase/releases/WS259	PRJEB513	O_volvulus_Cameroon_v3	12225	36
Oscheius tipulae	V	caenorhabditis.org	http://download.caenorhabditis.org/v1/sequence/	CEW1_nOt2	nOt.2.0	14938	43
Panagrellus redivivus	IV	wormbase.org	ftp://ftp.wormbase.org/pub/wormbase/releases/WS259	PRJNA186477	Pred3	26372	40
Parastrongyloides trichosuri	IV	parasite.wormbase.org	ftp://ftp.ebi.ac.uk/pub/databases/wormbase/parasite/releases/WBPS9	PRJEB515	P_trichosuri_KNP_v2_0_4	15010	31
Pristionchus exspectatus	V	pristionchus.org	http://pristionchus.org/download/	exspectatus_ augustus_prediction2013	P_exspectatus_v1	29247	34
Pristionchus pacificus	V	pristionchus.org	http://pristionchus.org/download/	pacificus_ Hybrid2_annotations	Hybrid2	27278	23
Rhabditophanes sp. kr3021	IV	parasite.wormbase.org	ftp://ftp.ebi.ac.uk/pub/databases/wormbase/parasite/releases/WBPS9	PRJEB1297	Rhabditophanes_sp_KR3021_v2_0_4	13496	26
Romanomermis culicivorax	I	parasite.wormbase.org	ftp://ftp.ebi.ac.uk/pub/databases/wormbase/parasite/releases/WBPS9	PRJEB1358	nRc.2.0	48376	15

Steinernema carpocapsae	IV	parasite.wormbase.org	ftp://ftp.ebi.ac.uk/pub/databases/wormbase/parasite/releases/WBPS9	PRJNA202318	S_carpo_v1_submitted	31944	46
Steinernema feltiae	IV	parasite.wormbase.org	ftp://ftp.ebi.ac.uk/pub/databases/wormbase/parasite/releases/WBPS9	PRJNA204661	S_felt_v1_submitted	36434	45
Steinernema glaseri	IV	parasite.wormbase.org	ftp://ftp.ebi.ac.uk/pub/databases/wormbase/parasite/releases/WBPS9	PRJNA204943	S_glas_v1_submitted	37119	40
Steinernema monticolum	IV	parasite.wormbase.org	ftp://ftp.ebi.ac.uk/pub/databases/wormbase/parasite/releases/WBPS9	PRJNA205067	S_monti_v1_submitted	38381	39
Steinernema scapterisci	IV	parasite.wormbase.org	ftp://ftp.ebi.ac.uk/pub/databases/wormbase/parasite/releases/WBPS9	PRJNA204942	S_scapt_v1_submitted	33149	46
Strongyloides papillosus	IV	parasite.wormbase.org	ftp://ftp.ebi.ac.uk/pub/databases/wormbase/parasite/releases/WBPS9	PRJEB525	S_papillosus_LIN_v2_1_4	18456	29
Strongyloides ratti	IV	wormbase.org	ftp://ftp.wormbase.org/pub/wormbase/releases/WS259	PRJEB125	S_ratti_ED321_v5_0_4	12483	31
Strongyloides stercoralis	IV	parasite.wormbase.org	ftp://ftp.ebi.ac.uk/pub/databases/wormbase/parasite/releases/WBPS9	PRJEB528	S_stercoralis_PV0001_v2_0_4	13098	28
Strongyloides venezuelensis	IV	parasite.wormbase.org	ftp://ftp.ebi.ac.uk/pub/databases/wormbase/parasite/releases/WBPS9	PRJEB530	S_venezuelensis_HH1_v2_0_4	16904	25
Toxocara canis	III	parasite.wormbase.org	ftp://ftp.ebi.ac.uk/pub/databases/wormbase/parasite/releases/WBPS9	PRJNA248777	Toxocara_canis_adult_r1.0	18596	58
Trichinella britovi	I	parasite.wormbase.org	ftp://ftp.ebi.ac.uk/pub/databases/wormbase/parasite/releases/WBPS9	PRJNA257433	T3_ISS120_r1.0	20907	20
Trichinella murrelli	I	parasite.wormbase.org	ftp://ftp.ebi.ac.uk/pub/databases/wormbase/parasite/releases/WBPS9	PRJNA257433	T5_ISS417_r1.0	18645	19
Trichinella nativa	I	parasite.wormbase.org	ftp://ftp.ebi.ac.uk/pub/databases/wormbase/parasite/releases/WBPS9	PRJNA257433	T2_ISS10_r1.0	17293	22
Trichinella nelsoni	I	parasite.wormbase.org	ftp://ftp.ebi.ac.uk/pub/databases/wormbase/parasite/releases/WBPS9	PRJNA257433	T7_ISS37_r1.0	17008	21
Trichinella papuae	I	parasite.wormbase.org	ftp://ftp.ebi.ac.uk/pub/databases/wormbase/parasite/releases/WBPS9	PRJNA257433	T10_ISS1980_r1.0	16247	22
Trichinella patagoniensis	I	parasite.wormbase.org	ftp://ftp.ebi.ac.uk/pub/databases/wormbase/parasite/releases/WBPS9	PRJNA257433	T12_ISS2496_r1.0	19538	21
Trichinella pseudospiralis T4.1	I	parasite.wormbase.org	ftp://ftp.ebi.ac.uk/pub/databases/wormbase/parasite/releases/WBPS9	PRJNA257433	T4_ISS13_r1.0	17161	22

Trichinella pseudospiralis T4.2	I	parasite.wormbase.org	ftp://ftp.ebi.ac.uk/pub/databases/wormbase/parasite/releases/WBPS9	PRJNA257433	T4_ISS588_r1.0	17158	20
Trichinella pseudospiralis T4.3	I	parasite.wormbase.org	ftp://ftp.ebi.ac.uk/pub/databases/wormbase/parasite/releases/WBPS9	PRJNA257433	T4_ISS176_r1.0	16961	18
Trichinella pseudospiralis T4.4	I	parasite.wormbase.org	ftp://ftp.ebi.ac.uk/pub/databases/wormbase/parasite/releases/WBPS9	PRJNA257433	T4_ISS470_r1.0	14920	23
Trichinella pseudospiralis T4.5	I	parasite.wormbase.org	ftp://ftp.ebi.ac.uk/pub/databases/wormbase/parasite/releases/WBPS9	PRJNA257433	T4_ISS141_r1.0	16075	21
Trichinella sp. t6	I	parasite.wormbase.org	ftp://ftp.ebi.ac.uk/pub/databases/wormbase/parasite/releases/WBPS9	PRJNA257433	T6_ISS34_r1.0	19518	21
Trichinella sp. t8	I	parasite.wormbase.org	ftp://ftp.ebi.ac.uk/pub/databases/wormbase/parasite/releases/WBPS9	PRJNA257433	T8_ISS272_r1.0	18455	20
Trichinella sp. t9	I	parasite.wormbase.org	ftp://ftp.ebi.ac.uk/pub/databases/wormbase/parasite/releases/WBPS9	PRJNA257433	T9_ISS409_r1.0	18558	19
Trichinella spiralis	I	parasite.wormbase.org	ftp://ftp.ebi.ac.uk/pub/databases/wormbase/parasite/releases/WBPS9	PRJNA257433	T1_ISS3_r1.0	19244	23
Trichinella zimbabwensis	I	parasite.wormbase.org	ftp://ftp.ebi.ac.uk/pub/databases/wormbase/parasite/releases/WBPS9	PRJNA257433	T11_ISS1029_r1.0	19269	21
Trichuris suis	I	parasite.wormbase.org	ftp://ftp.ebi.ac.uk/pub/databases/wormbase/parasite/releases/WBPS9	PRJNA208415	Tsuis_adult_male_1.0	14436	18
Wuchereria bancrofti	III	parasite.wormbase.org	ftp://ftp.ebi.ac.uk/pub/databases/wormbase/parasite/releases/WBPS9	PRJNA275548	Wb_PNG_Genome_assembly_pt22	11068	31

Supplementary Table 3: Phylogeny- versus sequence-based classification of nematode ZP modules. Phylogenetic and structural analysis of nematode ZP modules identified three major groups of modules with distinct ZP-C domain disulfide binding patterns (Types 1, 2, and 3), as shown in Figure 2 of the main text. Cohen et al. (2019 *Genetics* vol. 211) independently classified *C. elegans* ZP modules into groups based on the number of cysteine residues found within the ZP-C domain. The results of these two approaches are largely but not completely congruent, with disagreements occurring as a result of lineage-specific gains and losses of disulfides that alter the cysteine count.

	Module Classification	
**Protein**	**Present study (Phylogenetics and Homology Modelling)**	**Cohen et al. (Cysteine Counting)**
**CUTL-17**	Type 1	I (6 CYS)
**CUTL-18**	Type 1	I (6 CYS)
**CUTL-20a**	Type 1	I (6 CYS)
**CUTL-24b**	Type 1	II (4 CYS)
**CUTL-25**	Type 1	I (6 CYS)
**CUTL-26b**	Type 1	IIIb (8 CYS)
**CUTL-27**	Type 1	I (6 CYS)
**CUTL-28**	Type 1	II (4 CYS)
**CUTL-29**	Type 1	I (6 CYS)
**DYF-7**	Type 1	I (6 CYS)
**FBN-1a**	Type 1	IIIb (8 CYS)
**LET-653b**	Type 1	IIIb (8 CYS)
**NOAH-1c**	Type 1	I (6 CYS)
**NOAH-2**	Type 1	I (6 CYS)
**T23F1.5**	Type 1	I (6 CYS)
**CUT-6**	Type 2	IIIb (8 CYS)
**CUTL-5**	Type 2	IIIb (8 CYS)
**CUTL-6**	Type 2	IIIb (8 CYS)
**CUTL-7**	Type 2	IIIb (8 CYS)
**CUTL-8a**	Type 2	IIIb (8 CYS)
**CUTL-9**	Type 2	IIIb (8 CYS)
**CUTL-10**	Type 2	IIIb (8 CYS)
**CUTL-11**	Type 2	IIIb (8 CYS)
**CUTL-12a**	Type 2	IIIb (8 CYS)
**CUTL-13**	Type 2	IIIb (8 CYS)
**CUTL-16**	Type 2	IIIb (8 CYS)
**CUTL-19b**	Type 2	Not considered

**CUTL-22a**	Type 2	IIIb (8 CYS)
**CUTL-23a**	Type 2	IIIb (8 CYS)
**DPY-1a (M01E10.2)**	Type 2	IIIb (8 CYS)
**RAM-5**	Type 2	IIIb (8 CYS)
**CUT-1**	Type 3	IIIa (8 CYS)
**CUT-3**	Type 3	IIIa (8 CYS)
**CUT-4**	Type 3	IIIa (8 CYS)
**CUT-5b**	Type 3	IIIa (8 CYS)
**CUTL-1**	Type 3	IIIa (8 CYS)
**CUTL-2**	Type 3	IIIa (8 CYS)
**CUTL-3**	Type 3	IIIa (8 CYS)
**CUTL-4**	Type 3	IIIa (8 CYS)
**CUTL-14**	Type 3	IIIa (8 CYS)
**CUTL-15**	Type 3	IIIa (8 CYS)
**F46G11.6**	Type 3	IIIa (8 CYS)
**T01D1.8b**	Type 3	IIIa (8 CYS)
**R52.6**	Not considered	II (4 CYS)
**T01D1.8a**	Not considered	II (4 CYS)

Supplementary Table 4: Codon model analysis of standalone ZP-C domain subfamilies. Three models were fit to each data set: M0, M8a, and M8. Reported for each are the number of parameters (n.p.), the log-likelihood ($\log_e L$), relevant parameter estimates (the total tree length in substitutions per codon, the transition:transversion rate ratio (κ), and dN/dS distribution parameters (ω, *p*, *q*, p_β, and ω_P)), and the *P* value for the likelihood ratio test between models M8 and M8a (assuming 1 degree of freedom).

Subfamily	Model	n.p.	$\log_e L$	Tree Length	κ	dN/dS	LRT *P* value
CUTL-19	M0	47	-12939.866121	38.28212	1.28246	ω = 0.13478	—
	M8a	49	-12536.379917	49.23845	1.31699	*p* = 0.77247<br>*q* = 4.22570<br>p_β = 0.99104	—
	M8	50	-12536.379917	49.23844	1.31699	*p* = 0.77247<br>*q* = 4.22568<br>p_β = 0.99104<br>ω_P = 1.00000	1.00000
F46G11.6	M0	63	-9478.944527	42.11027	1.57335	ω = 0.09455	—
	M8a	65	-9135.95464	57.80803	1.72020	*p* = 0.58348<br>*q* = 4.53111<br>p_β = 0.99999	—
	M8	66	-9135.95464	57.80808	1.72020	*p* = 0.58348<br>*q* = 4.53115<br>p_β = 0.99999<br>ω_P = 1.00000	1.00000
T01D1.8	M0	87	-12031.667882	54.58330	1.63196	ω = 0.09392	—
	M8a	89	-11477.597608	73.90117	1.81456	*p* = 0.65778<br>*q* = 7.09069<br>p_β = 0.95636	—
	M8	90	-11477.597608	73.90173	1.81458	*p* = 0.65779<br>*q* = 7.09099<br>p_β = 0.95636<br>ω_P = 1.00000	1.00000

Supplementary Table 5: RaptorX homology modelling results of *C. elegans* ZPD proteins. The full-length protein was analyzed in almost all cases (*Length* and *Input range*); the standalone ZP-C domain proteins (CUTL-19b, F46G11.6, and T01D1.8b) were trimmed to remove predicted N- and C-terminal propeptide features before modelling, and the N-terminus of FBN-1a was trimmed to meet RaptorX input length requirements. Only models generated using the ZP module from human uromodulin (PDB 4wrn) were evaluated to facilitate comparisons among models. 4wrn-models were usually, but not always, the top ranked model; *Rank* refers to the rank assigned to the 4wrn-model by RaptorX. The next six columns (*P value, Score, uGDT, GDT, uSeqID, SeqID*) report various RaptorX modelling quality statistics. Finally, *ZP-N range* and *ZP-C range* indicate the approximate boundaries of the two domains (relative to the full-length sequence). These ranges reflect the RaptorX subject-template alignment and the domain boundaries reported for the template by Bokhove et al. (2016 *PNAS* vol. 113).

C. elegans Protein	Length (aa)	Input range	Rank	*P* value	Score	uGDT	GDT	uSeqID	SeqID	ZP-N range	ZP-C range
**CUT-1**	424	1:424	1	2.0E-08	150	126	45	34	12	28:125	149:279
**CUT-3**	389	1:389	1	3.5E-08	150	129	40	32	10	29:126	150:318
**CUT-4**	507	1:507	1	3.4E-09	158	131	43	31	10	38:133	157:302
**CUT-5b**	379	1:379	1	2.0E-08	155	128	42	29	9	28:127	151:303
**CUT-6**	572	1:572	1	3.2E-06	200	149	26	46	8	228:329	353:526
**CUTL-1**	399	1:399	1	8.9E-09	149	127	37	33	10	55:155	179:355
**CUTL-2**	382	1:382	1	1.1E-11	163	136	41	36	11	24:124	150:325
**CUTL-3**	405	1:405	1	1.9E-08	154	133	38	32	9	29:126	186:341
**CUTL-4**	469	1:469	2	4.5E-08	150	120	41	29	10	31:130	160:294
**CUTL-5**	437	1:437	2	2.5E-08	147	119	42	39	14	29:121	150:287
**CUTL-6**	374	1:374	1	1.2E-08	161	129	40	38	12	35:132	156:317
**CUTL-7**	585	1:585	1	1.3E-09	154	128	49	33	13	80:180	204:337
**CUTL-8a**	625	1:625	1	4.5E-09	159	119	41	33	11	29:129	153:292
**CUTL-9**	562	1:562	1	1.1E-07	138	118	46	32	13	NA[a]	335:490
**CUTL-10**	403	1:403	1	8.5E-09	161	129	39	35	10	31:131	155:331
**CUTL-11**	384	1:384	1	5.2E-08	160	116	36	37	11	34:135	159:318
**CUTL-12a**	569	1:569	1	1.9E-10	160	123	38	39	12	33:134	157:317
**CUTL-13**	445	1:445	2	1.5E-08	157	123	43	36	13	26:128	153:284
**CUTL-14**	270	1:270	2	4.9E-10	153	122	45	36	13	28:125	149:270
**CUTL-15**	385	1:385	1	8.2E-09	158	132	43	36	12	30:132	154:300
**CUTL-16**	488	1:488	2	1.7E-08	150	120	43	29	10	30:123	147:280
**CUTL-17**	912	1:912	1	4.3E-08	156	131	44	37	13	504:604	623:791
**CUTL-18**	801	1:801	1	2.0E-08	147	129	38	35	10	468:571	599:754
**CUTL-19b**	237	21:216	1	1.4E-06	89	83	42	23	12	NA	53:208
**CUTL-20a**	360	1:360	1	7.4E-10	142	123	34	25	7	25:119	150:314
**CUTL-22a**	445	1:445	1	1.0E-08	152	131	43	41	14	58:157	187:351
**CUTL-23a**	773	1:773	2	1.2E-09	148	127	49	40	16	449:546	562:699

**CUTL-24b**	601	1:601	1	1.2E-05	104	154	26	41	7	30:129	393:548
**CUTL-25**	385	1:385	1	1.0E-08	162	137	35	30	8	31:131	157:338
**CUTL-26b**	502	1:502	1	1.7E-07	144	126	44	33	11	29:122	135:283
**CUTL-27**	969	1:969	1	2.4E-08	146	127	45	36	13	624:724	750:896
**CUTL-28**	696	1:696	1	2.5E-09	159	124	33	36	10	282:381	419:589
**CUTL-29**	390	1:390	1	2.2E-08	155	137	43	43	13	22:118	152:318
**DPY-1a**	1387	1:1387	2	3.4E-06	191	151	29	47	9	1001:1103	1132:1295
**DYF-7**	446	1:446	1	1.5E-08	164	143	41	44	13	26:141	165:341
**F46G11.6**	160	21:160	1	3.3E-06	90	77	55	21	15	NA	38:160
**FBN-1a**	2779	280:2779	1	4.1E-09	155	146	43	47	14	2420:2528	2555:2713
**LET-653b**	653	1:653	1	5.0E-10	134	140	31	42	9	212:316	440:597
**NOAH-1c**	1052	1:1052	1	4.1E-08	147	131	37	48	14	632:773	804:976
**NOAH-2**	741	1:741	1	4.1E-09	163	133	38	39	11	386:484	515:674
**RAM-5**	711	1:711	2	1.5E-10	156	125	46	30	11	33:129	141:271
**T01D1.8b**	189	26:189	1	3.0E-06	94	89	54	20	12	NA	47:189
**T23F1.5**	1262	1:1262	2	3.4E-09	153	116	43	24	9	911:1007	1039:1172

a — Modelling of the *C. elegans* CUTL-9 ZP-N domain failed due to the presence of a long insertion within the DE loop. Homology modelling of CUTL-9 subfamily members (i.e., orthologs from other species) that lack the insertion successfully recovered the ZP-N domain (results not shown).

Supplementary Figures

Supplementary Figure 1: Positional map between trimmed and untrimmed versions of *C. elegans* CUT-1. Shown here is an alignment between *C. elegans* CUT-1 (numbered according to sequence position) and a trimmed version of CUT-1 extracted from GISMO alignment #38, the alignment replicate with the highest log-likelihood ratio score (numbered according to alignment column). Lowercase residues in the original CUT-1 sequence indicate positions that were trimmed away during alignment estimation (represented by dashes in the corresponding trimmed sequence); the retained positions comprise the inferred core alignment (including a gap of one residue that was inferred within the CUT-1 core at alignment column 236). Shown above the original CUT-1 sequence are (1) the predicted signal peptide (s), (2) the predicted R/K cleavage cut-site (X), and the interdomain linker (I).

```
                   ssssssssssssssssss
 AA sequence #     1.......10.......20.......30.......40.......50.......60.......70.......80
CUT-1-ORIGINAL     mtwkpiiclaalvlsasaipvdnnvegepeveCGPNSITVNFNTRNPFEGHVYYKGLYDQagCRSDEGGRQVAGIELPFD
CUT-1-ALI38        --------------------------------CGPNSITVNFNTRNPFEGHVYYKGLYDQ--CRSDEGGRQVAGIELPFD
 Alignment #                                       1.......10.......20........  30.......40......

                                                               IIIII
                   ........90......100......110......120......130......140......150......160
                   SCNTARTRSLNpkgvfvsTTVVISFHPQFVTKVDRAYRIQCFYMESDKTvStqievsDLTTAFQTQVVPMPVCKYEILDG
                   SCNTARTRSLN-------TTVVISFHPQFVTKVDRAYRIQCFYMESDKT-S------DLTTAFQTQVVPMPVCKYEILDG
                   ..50.......       .60.......70.......80........ .     90......100......110..

                   .......170......180......190......200......210......220......230......240
                   GPSGQPIQFATIGQQVYHKWTCDSEttdTFCAVVHSCTVDDGNGDTVQILNEEGCALDKFLLNNLEYPTDLMAGQEAHVY
                   GPSGQPIQFATIGQQVYHKWTCDSE---TFCAVVHSCTVDDGNGDTVQILNEEGCALDKFLLNNLEYPTDLMAGQEAHVY
                   .....120......130.......   140......150......160......170......180.........

                   .......250......260......270......280......290......300......310......320
                   KYADRSQLFYQCQISITIKdpgsECARPTCSEPQgfgavkqagaggahaaaapqagveevqaapvaaaapvaapvaaaaa
                   KYADRSQLFYQCQISITIK----ECARPTCSEPQ----------------------------------------------
                 190......200.......   210.........

                                     X
                   .......330......340......350... ...360......370......380......390........
                   apavpraTlaqlrllRKKRSFGEnegILDVRVE-INTLDIMEGASPSAPEaaalvseesvrrratstgicltpigfasfl
                   -------T-------RKKRSFGE---ILDVRVE?INTLDIMEGASPSAPE------------------------------
                        220       ........  230......240......250..

                 400......410......420....  (424 AA residues)
                   gigtivatalsatifyvarptshkh
                   -------------------------
                                            (252 Alignment columns)
```

Supplementary Figure 2: Branch support metrics for the majority rule consensus tree. (a) Branch Recovery Proportions (BRP) and (b) Transfer Bootstrap Expectations (TBE) for the nematode ZP module majority rule consensus phylogeny. (c) Overlapping histograms showing the distribution of BRP and TBE support values for the entire tree. By definition, no branches have support values below 50% in the majority rule tree.

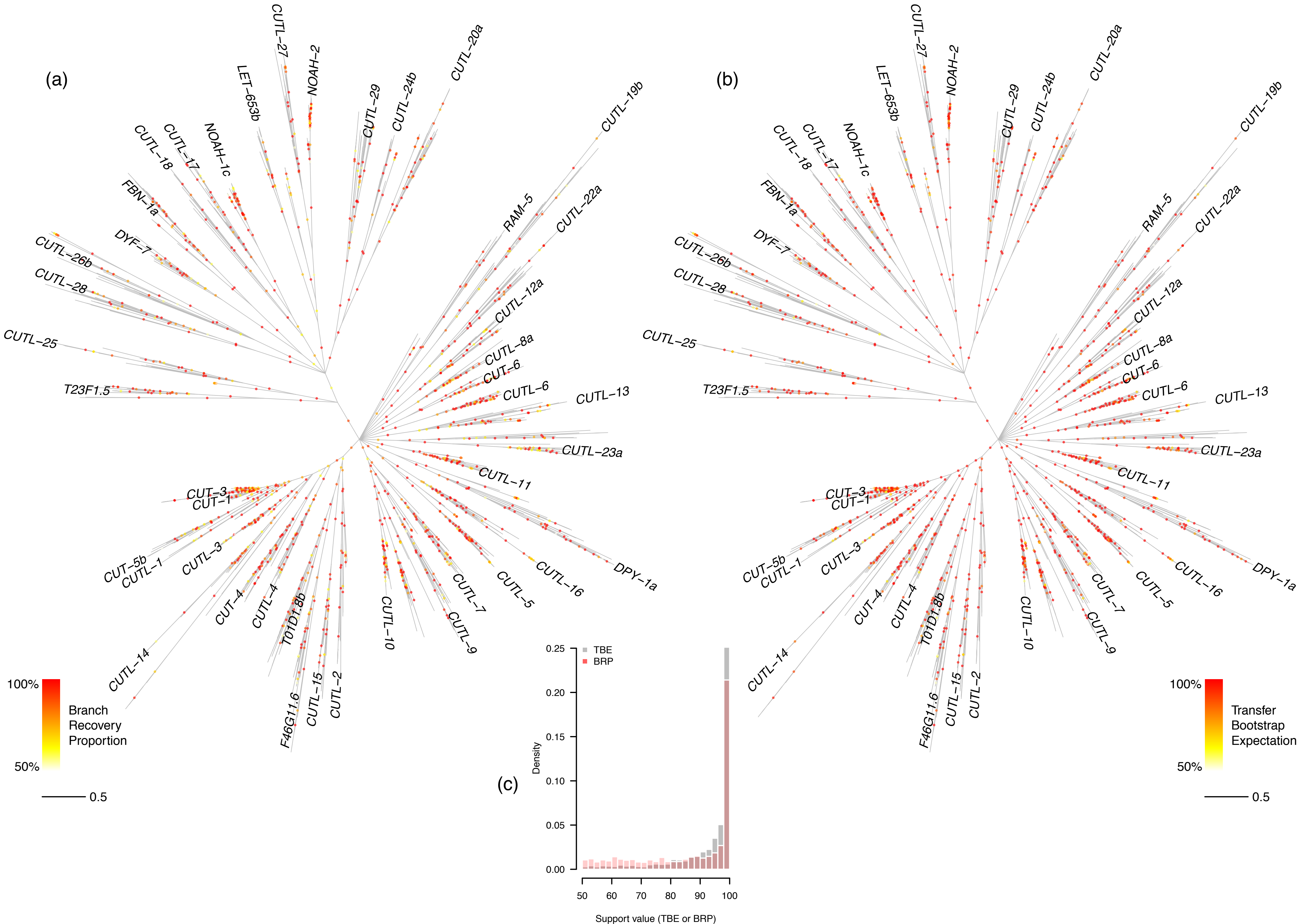
(a)
(b)
(c)
CUTL–27
NOAH–2
CUTL–20a
LET–653b
CUTL–29
CUTL–24b
CUTL–19b
CUTL–18
CUTL–17
NOAH–1c
FBN–1a
RAM–5
CUTL–22a
DYF–7
CUTL–26b
CUTL–28
CUTL–12a
CUTL–25
CUTL–8a
CUT–6
CUTL–6
T23F1.5
CUTL–13
CUTL–23a
CUTL–11
CUT–3
CUT–1
CUT–5b
CUTL–1
CUTL–3
CUT–4
CUTL–4
T01D1.8b
CUTL–10
CUTL–7
CUTL–5
CUTL–16
DPY–1a
CUTL–9
CUTL–14
F46G11.6
CUTL–15
CUTL–2
100%
50%
Branch Recovery Proportion
Transfer Bootstrap Expectation
0.5
TBE
BRP
Density
Support value (TBE or BRP)
0.00
0.05
0.10
0.15
0.20
0.25
50
60
70
80
90
100

Supplementary Figure 3: Branch support metrics for the focal alignment's maximum likelihood tree. (a) Branch Recovery Proportions (BRP) and (b) Transfer Bootstrap Expectations (TBE) for the nematode ZP module phylogeny inferred using the focal alignment (the top scoring alignment according to LLR score). The labelled arrows on (a) denote the Minimal Ancestor Deviation (MAD) root and the phylogenetic midpoint. Also shown in (a) are the phylogenetic boundaries between the Type 1, 2, and 3 ZP modules. Type 1 modules are paraphyletic, given the MAD root; Type 2 and 3 modules together form a monophyletic group, within which the Type 3 modules form their own monophyletic clade. (c) Overlapping histograms showing the distribution of BRP and TBE support values for the entire tree.

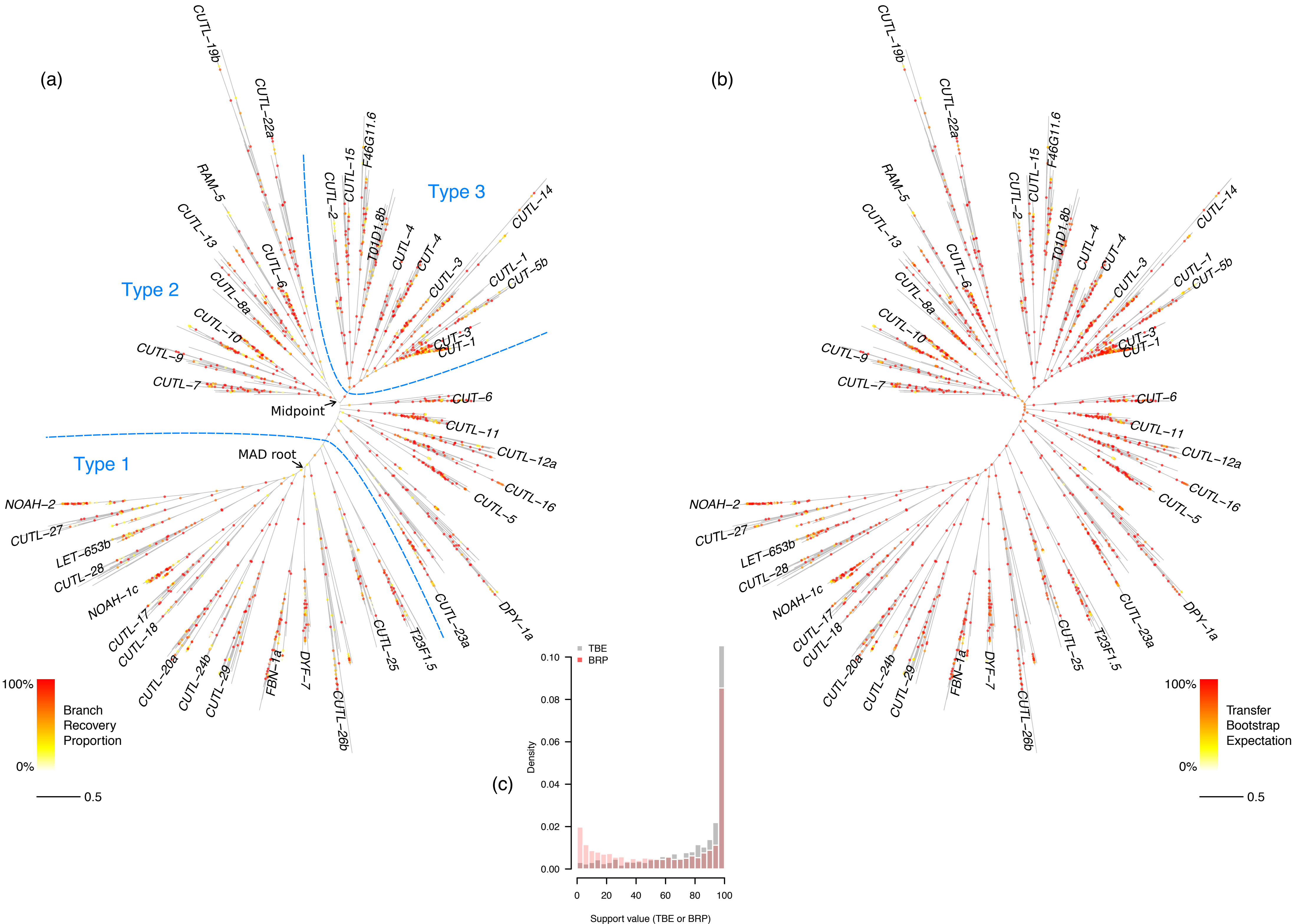
(a)
(b)
(c)
Type 1
Type 2
Type 3
Midpoint
MAD root
Branch
Recovery
Proportion
Transfer
Bootstrap
Expectation
100%
0%
0.5
CUTL−19b
CUTL−22a
RAM−5
CUTL−13
CUTL−6
CUTL−8a
CUTL−10
CUTL−9
CUTL−7
CUTL−2
CUTL−15
F46G11.6
T01D1.8b
CUTL−4
CUT−4
CUTL−3
CUTL−1
CUT−5b
CUTL−14
CUT−3
CUT−1
CUT−6
CUTL−11
CUTL−12a
CUTL−16
CUTL−5
DPY−1a
CUTL−23a
T23F1.5
CUTL−25
CUTL−26b
DYF−7
FBN−1a
CUTL−29
CUTL−24b
CUTL−20a
CUTL−18
CUTL−17
NOAH−1c
CUTL−28
LET−653b
CUTL−27
NOAH−2
TBE
BRP
Density
Support value (TBE or BRP)

Supplementary Figure 4: Variable amino acid conservation patterns among nematode ZP modules. Amino acid conservation patterns in phylogenetically-defined subsets of the top-scoring nematode ZP module alignment, shown via sequence logos: (a) Type 1 modules, (b) Type 2 modules, (c) Type 3 modules. The height of each amino acid indicates its prevalence at the given position in the subsetted alignment. Connections between cysteine residues indicate inferred disulfide linkages; also shown are the approximate boundaries of the ZP-N and ZP-C domains, the internal and external hydrophobic patches (IHP/EHP), and the consensus cleavage site (CCS). The sequence logo for the entire data set is shown in Figure 1.

(a) Type 1

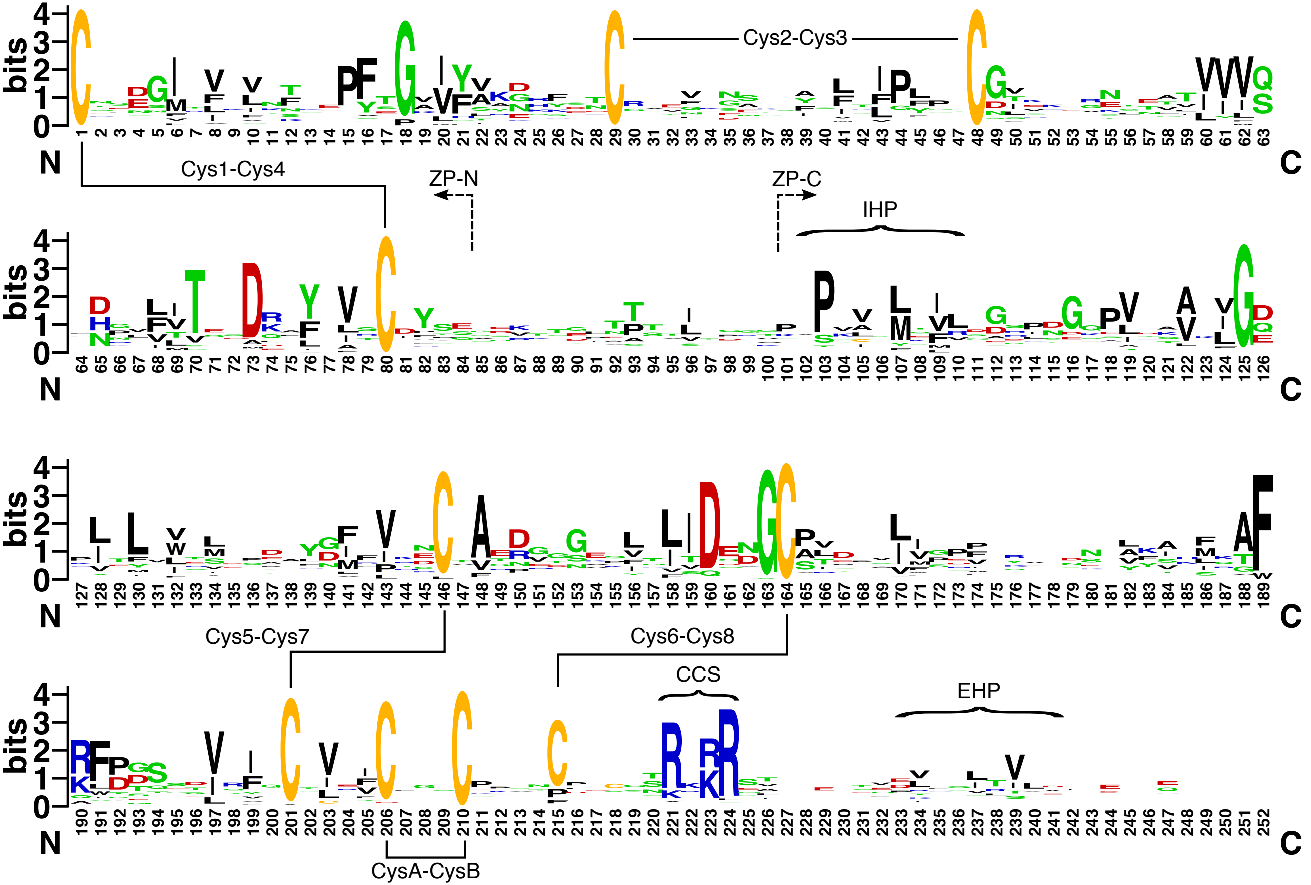

bits
N
C
Cys2-Cys3
Cys1-Cys4
ZP-N
ZP-C
IHP
Cys5-Cys7
Cys6-Cys8
CCS
EHP
CysA-CysB

(b) Type 2

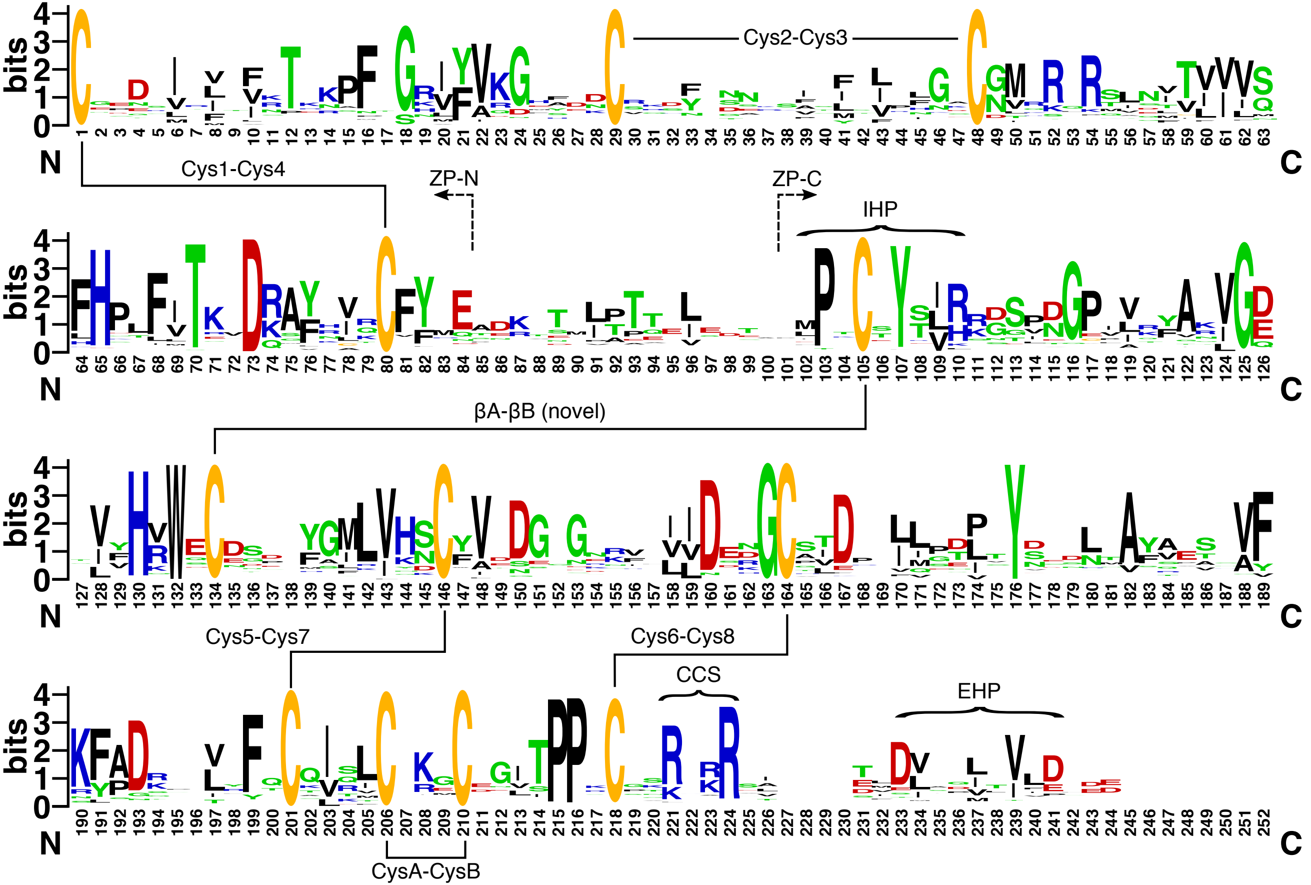
bits
N
C
Cys2-Cys3
Cys1-Cys4
ZP-N
ZP-C
IHP
βA-βB (novel)
Cys5-Cys7
Cys6-Cys8
CCS
EHP
CysA-CysB

(c) Type 3

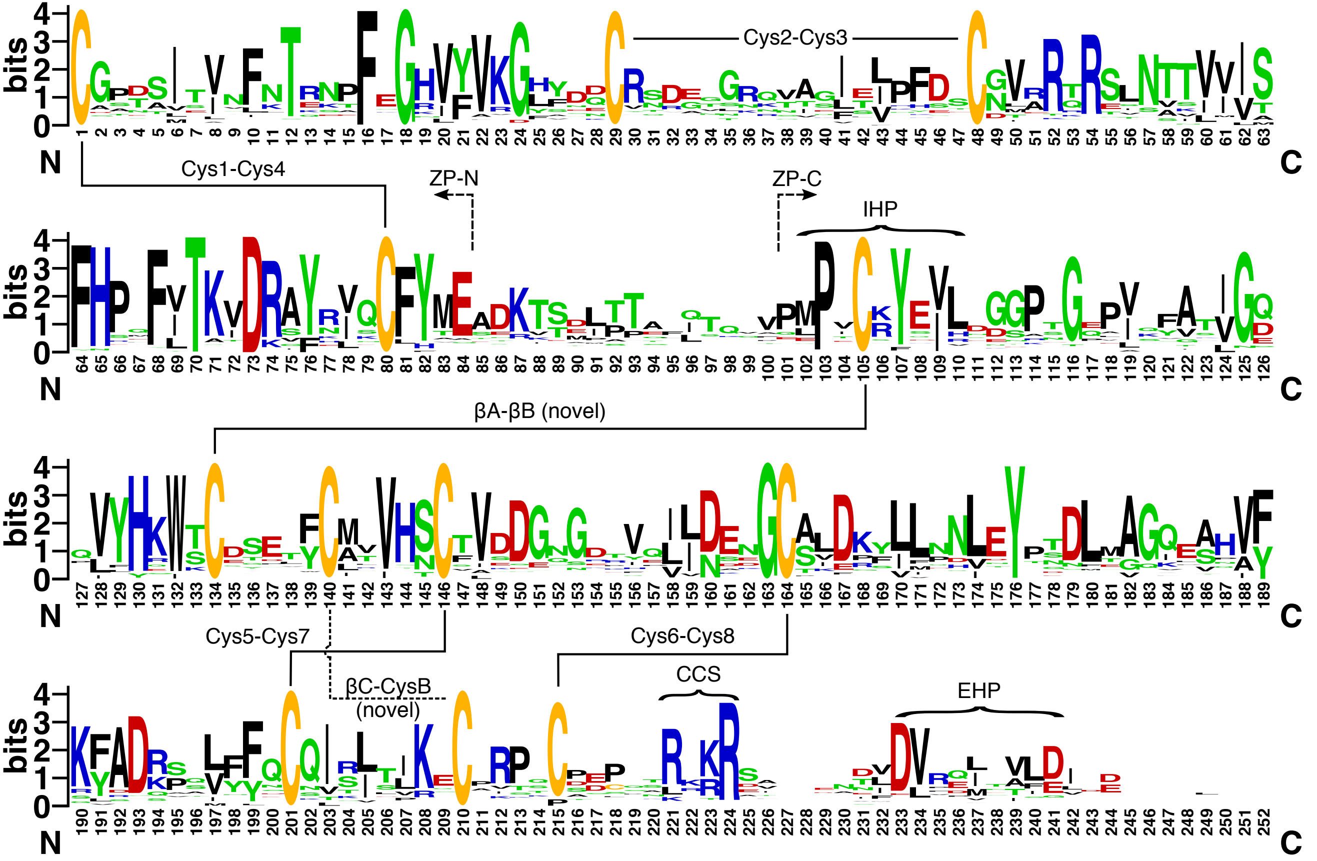
bits
N
C
Cys2-Cys3
Cys1-Cys4
ZP-N
ZP-C
IHP
βA-βB (novel)
Cys5-Cys7
Cys6-Cys8
βC-CysB
(novel)
CCS
EHP

Supplementary Figure 5: Minimal Ancestor Deviation (MAD) rooting of the nematode ZP module phylogeny. (a) Majority rule consensus tree. (b) Fully resolved tree obtained using the focal alignment. Branches are colour-coded by the degree of deviation from clock-like expectation induced by assuming a root on the given branch (AD; see legend). Bars indicate the inferred origins of Type 1, 2, and 3 ZP-C domain cysteine connectivity patterns (Figure 2b). The arrows denote the phylogenetic midpoints. Despite considerable uncertainty in the precise location of the root node, the results strongly point to the root falling somewhere within the Type 1 portion of the phylogeny.

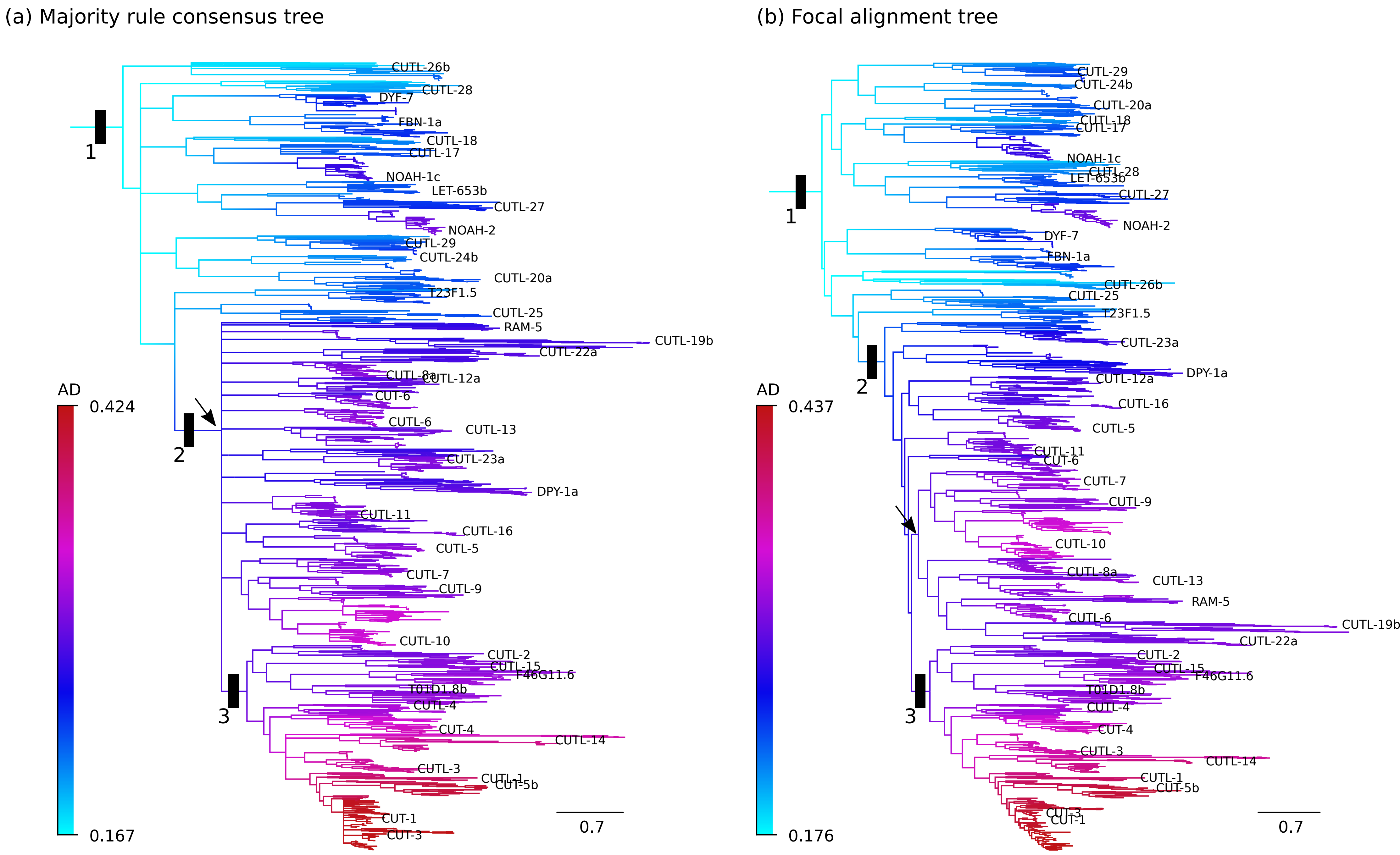
(a) Majority rule consensus tree
CUTL-26b
CUTL-28
DYF-7
FBN-1a
CUTL-18
CUTL-17
NOAH-1c
LET-653b
CUTL-27
NOAH-2
CUTL-29
CUTL-24b
CUTL-20a
T23F1.5
CUTL-25
RAM-5
CUTL-19b
CUTL-22a
CUTL-8a
CUTL-12a
CUT-6
CUTL-6
CUTL-13
CUTL-23a
DPY-1a
CUTL-11
CUTL-16
CUTL-5
CUTL-7
CUTL-9
CUTL-10
CUTL-2
CUTL-15
F46G11.6
T01D1.8b
CUTL-4
CUT-4
CUTL-14
CUTL-3
CUTL-1
CUT-5b
CUT-1
CUT-3
1
2
3
AD
0.424
0.167
0.7
(b) Focal alignment tree
CUTL-29
CUTL-24b
CUTL-20a
CUTL-18
CUTL-17
NOAH-1c
CUTL-28
LET-653b
CUTL-27
NOAH-2
DYF-7
FBN-1a
CUTL-26b
CUTL-25
T23F1.5
CUTL-23a
DPY-1a
CUTL-12a
CUTL-16
CUTL-5
CUTL-11
CUT-6
CUTL-7
CUTL-9
CUTL-10
CUTL-8a
CUTL-13
RAM-5
CUTL-6
CUTL-19b
CUTL-22a
CUTL-2
CUTL-15
F46G11.6
T01D1.8b
CUTL-4
CUT-4
CUTL-3
CUTL-14
CUTL-1
CUT-5b
CUT-3
CUT-1
1
2
3
AD
0.437
0.176
0.7

Supplementary Figure 6: Phylogenetic distribution of ZP modules across the major nematode clades. The majority rule consensus tree of Figure 2 (main text) was redrawn to show only the ZP modules from *C. elegans* (a Clade V species) and representative species from the three other major nematode clades covered in the present study (three from Clade I, two from Clade III, and three from Clade IV). Protein names are shown only for *C. elegans* ZP modules; for the other species, only the clade and species IDs are shown (see legend). Clade designations follow Blaxter et al. (1998 *Nature* vol. 392); the four relevant groups are related as follows: (((Clade V, Clade IV), Clade III), Clade I). The broad distribution of ZP modules observed for each of the clades indicates that the duplication events that generated nematode ZP module diversity largely predate the speciation events that gave rise to the major nematode groups.

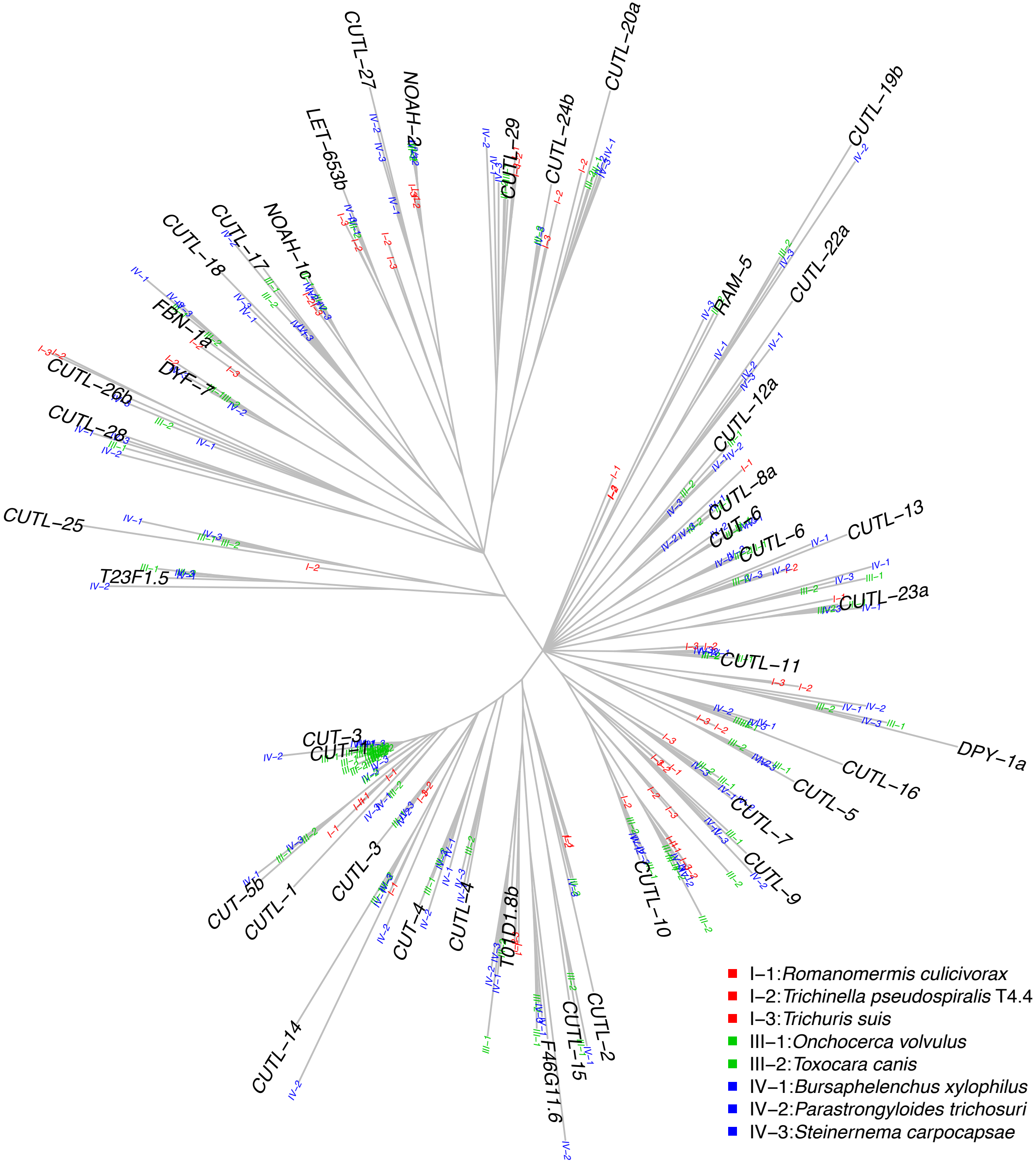
CUTL–27
NOAH–2
LET–653b
CUTL–29
CUTL–24b
CUTL–20a
CUTL–19b
CUTL–22a
RAM–5
CUTL–12a
CUTL–8a
CUT–6
CUTL–6
CUTL–13
CUTL–23a
CUTL–11
DPY–1a
CUTL–16
CUTL–5
CUTL–7
CUTL–9
CUTL–10
CUTL–2
CUTL–15
F46G11.6
T01D1.8b
CUTL–4
CUT–4
CUTL–14
CUTL–3
CUTL–1
CUT–5b
CUT–3
CUT–1
T23F1.5
CUTL–25
CUTL–28
CUTL–26b
DYF–7
FBN–1a
CUTL–18
CUTL–17
NOAH–1c
I–1: Romanomermis culicivorax
I–2: Trichinella pseudospiralis T4.4
I–3: Trichuris suis
III–1: Onchocerca volvulus
III–2: Toxocara canis
IV–1: Bursaphelenchus xylophilus
IV–2: Parastrongyloides trichosuri
IV–3: Steinernema carpocapsae

Supplementary Figure 7: Phylogenetic distribution of nematode ZPD protein domain architecture. ZPD proteins often contain additional domains upstream of the ZP module. The most commonly observed Pfam domain predictions (not including 'ZP domain' predictions, which were unsurprisingly the most frequent) were mapped onto the ZP module majority rule consensus tree of Figure 2: green circles = Epidermal Growth Factor (EGF)-like domains; purple crosses = von Willebrand factor type A (vWFA) domains; orange triangles = PAN domains. Tip names are shown only for *C. elegans* ZP modules, with those that possess upstream domains (according to domain annotations on WormBase.org) labelled accordingly.

EGF-like domains were predicted only within the FBN-1 subfamily. vWFA domains were found in the CUT-6, CUTL-23, and DPY-1 subfamilies, as well as in the sister group to the CUTL-23 subfamily (which lacks a *C. elegans* ortholog) and a rogue ZP module from a Clade I nematode. PAN domains were distributed across seven Type 1 subfamilies (CUTL-17, CUTL-18, CUTL-27, CUTL-28, NOAH-1, NOAH-2, and LET-653), consistent with *C. elegans*-based expectations, but were also observed for Clade I nematode ZP modules within the DYF-7 subfamily; subsequent examination showed that this unusual finding was due to the artefactual fusion of CUTL-28 homologs (which possess PAN domains) to DYF-7 homologs (which do not) within Clade I nematodes (results not shown). Assuming false negatives are more plausible than recurrent domain losses within subfamilies, the overall pattern suggests deep conservation of domain architecture.

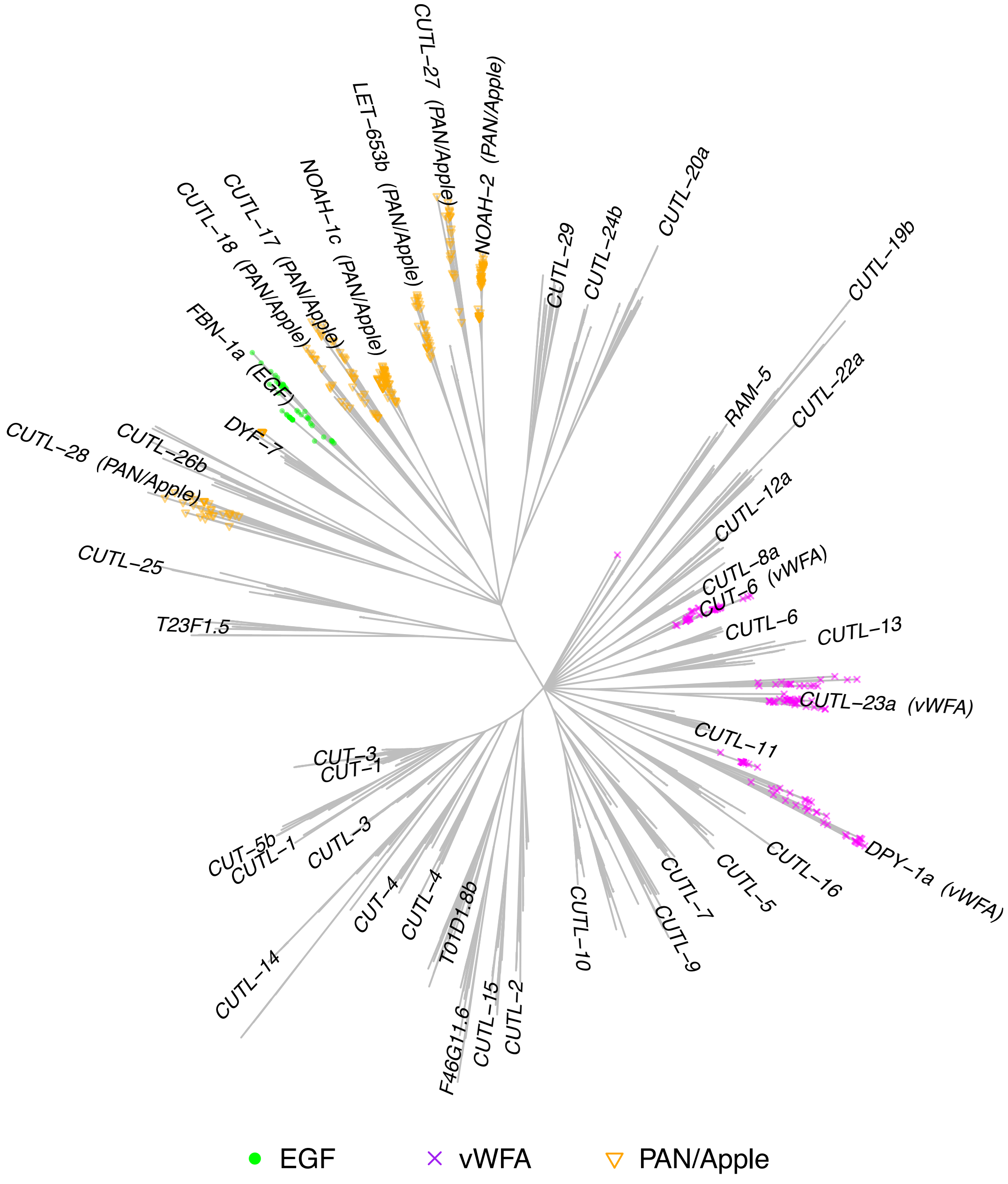

CUTL–27 (PAN/Apple)
NOAH–2 (PAN/Apple)
LET–653b (PAN/Apple)
NOAH–1c (PAN/Apple)
CUTL–17 (PAN/Apple)
CUTL–18 (PAN/Apple)
FBN–1a (EGF)
DYF–7
CUTL–26b
CUTL–28 (PAN/Apple)
CUTL–25
T23F1.5
CUTL–29
CUTL–24b
CUTL–20a
CUTL–19b
CUTL–22a
RAM–5
CUTL–12a
CUTL–8a
CUT–6 (vWFA)
CUTL–6
CUTL–13
CUTL–23a (vWFA)
CUTL–11
DPY–1a (vWFA)
CUTL–16
CUTL–5
CUTL–7
CUTL–9
CUTL–10
CUTL–2
CUTL–15
F46G11.6
T01D1.8b
CUTL–4
CUT–4
CUTL–14
CUTL–3
CUT–5b
CUTL–1
CUT–3
CUT–1
EGF
vWFA
PAN/Apple

Supplementary Figure 8: Selective constraint profiles for standalone ZP-C proteins. Site-specific estimates of selective constraint (dN/dS) for (a) the T01D1.8 subfamily, (b) the F46G11.6 subfamily, and (c) the CUTL-19 subfamily. The y-axes shows posterior mean estimates of dN/dS (± standard errors) obtained using the M8 codon substitution model, while the x-axes shows sequence position (annotated with secondary structure predictions for the *C. elegans* member of the respective subfamily; obtained from RaptorX ss3 secondary structure predictions, given untrimmed sequences as input). Orange dashes indicate cysteine residues in the *C. elegans* sequence; selected cysteines are labelled, following Figure 1 of the main text. Gaps in the dN/dS plot reflect sequence regions that were trimmed during alignment.

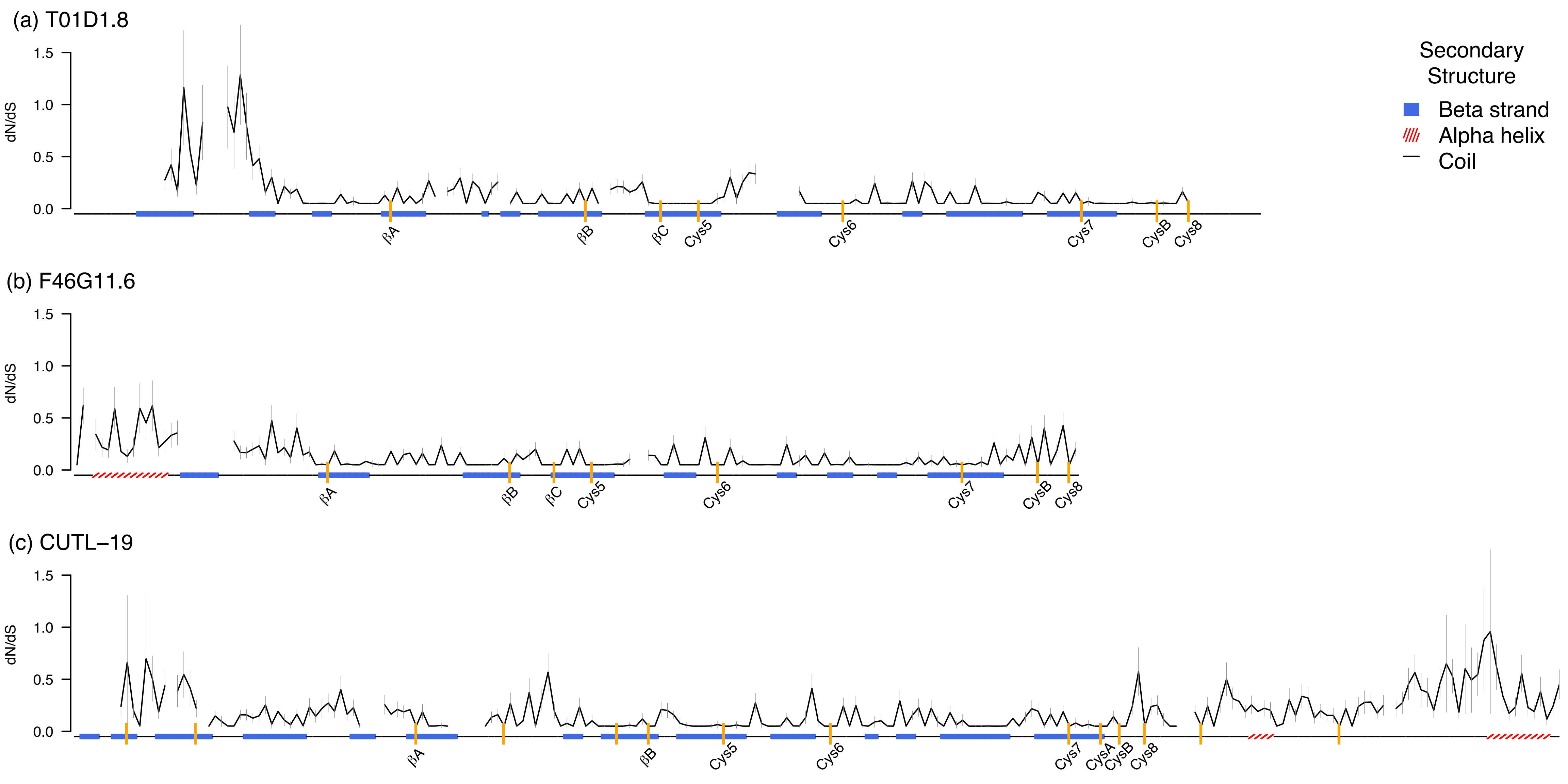

(a) T01D1.8
(b) F46G11.6
(c) CUTL−19
dN/dS
0.0
0.5
1.0
1.5
βA
βB
βC
Cys5
Cys6
Cys7
CysA
CysB
Cys8
Secondary Structure
Beta strand
Alpha helix
Coil

Supplementary Figure 9: Conserved disulfide connectivity patterns in nematode ZP-N domains. ZP-N domains from *C. elegans* ZP module homology models (pink lines) were aligned and superimposed on the template structure, human uromodulin (grey cartoon). Two orientations are shown. Cysteine residues in the *C. elegans* ZP-N domains are shown in stick format, and the two disulfide linkages typical of ZP-N domains (Cys1-Cys4 and Cys2-Cys3) are shown for the template as grey dot clouds. These disulfides were recovered in all models, with the exception of the three short ZPD proteins (which lack ZP-N domains) and CUTL-9 (which possesses a large insertion that disrupted homology modelling of the ZP-N domain; not shown). Additionally, a pair of cysteines that evolved within the CUTL-5 subfamily are well positioned to form a novel disulfide between βF (position 59) and βG (position 77).

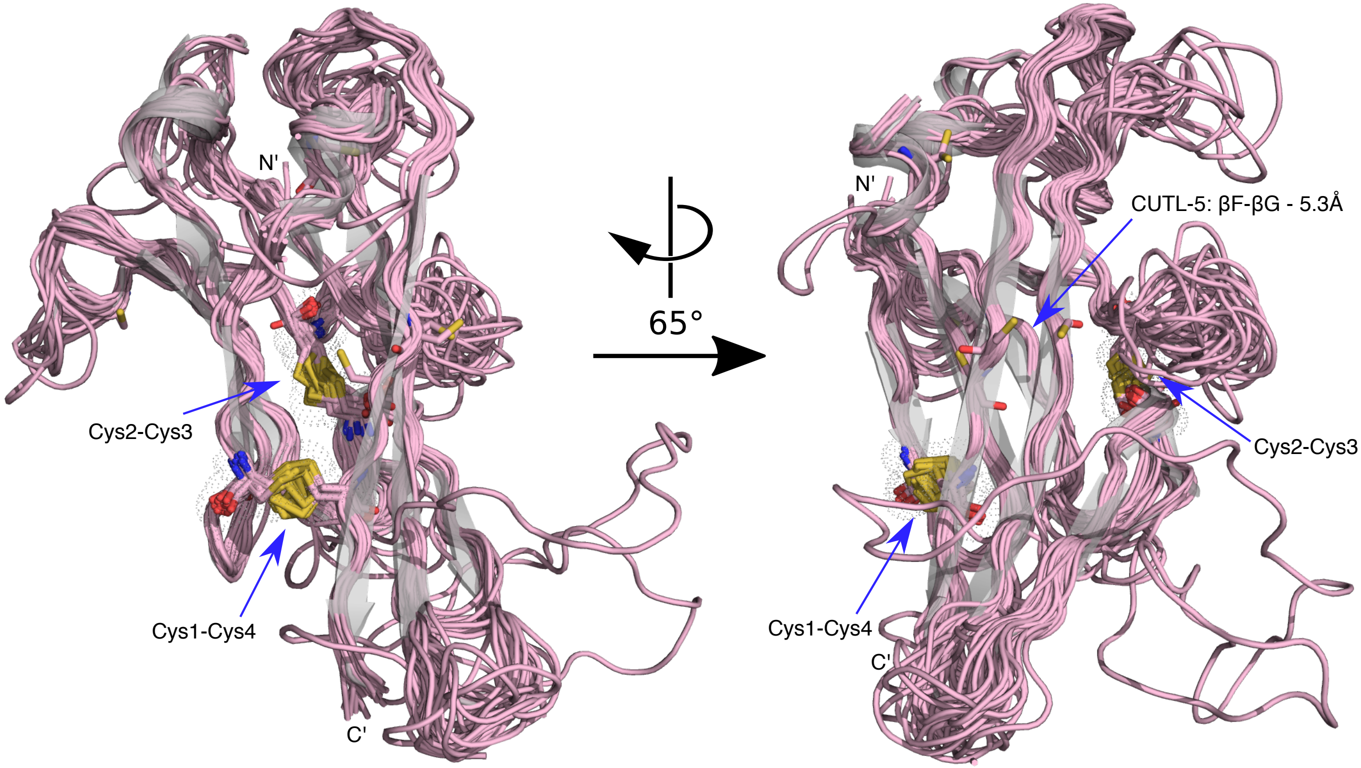
N'
Cys2-Cys3
Cys1-Cys4
C'
65°
N'
CUTL-5: βF-βG - 5.3Å
Cys2-Cys3
Cys1-Cys4
C'

Supplementary Figure 10: Variable disulfide connectivity patterns in nematode ZP-C domains. ZP-C domains from *C. elegans* ZP module homology models (pink lines) were aligned and superimposed on the template structure, human uromodulin (grey cartoon). Two orientations are shown, and models are grouped by ZP-C domain type: (a) Type 1; (b) Type 2; (c) Type 3. Cysteine residues in the *C. elegans* ZP-C domains are shown in stick format, and the three disulfide linkages typical of ZP-C domains (Cys5-Cys7, Cys6-Cys8, and CysA-CysB) are shown for the template as grey dot clouds. C-terminal tails (mid-FG loop onward) were often quite long and disordered, if modelled at all, and were therefore removed for clarity. Four patterns are apparent:

1. The Cys5-Cys7 disulfide is conserved across all models, with the exception of CUTL-28b.
2. The Cys6-Cys8 disulfide appears to be broadly conserved but modelling and alignment uncertainty make this conclusion tentative for Type 1 domains; the expected Cys6-Cys8 linkage was supported for some Type 1 models but atypical Cys6-CysB and CysA-Cys8 linkages were observed for others. These binding patterns leave the expected partners unbound and distant from one another, suggesting that they may simply reflect modelling uncertainty in the largely disordered FG loop.
3. The CysA-CysB disulfide is conserved in Type 2 ZP-C domains, variable in Type 1 domains (recovered in two models, lost in one (CUTL-24b), but uncertain in most; see point 2, above), and modified in Type 3 domains, where CysA has been lost and replaced with a novel cysteine in the adjacent βC strand.
4. Cysteines in βA (position 105) and βB (position 134) define a novel disulfide specific to Type 2 and 3 ZP-C domains. A partially similar disulfide is inferred in FBN-1a (a Type 1 domain) between βA (position 105, again) and βF (position 203).

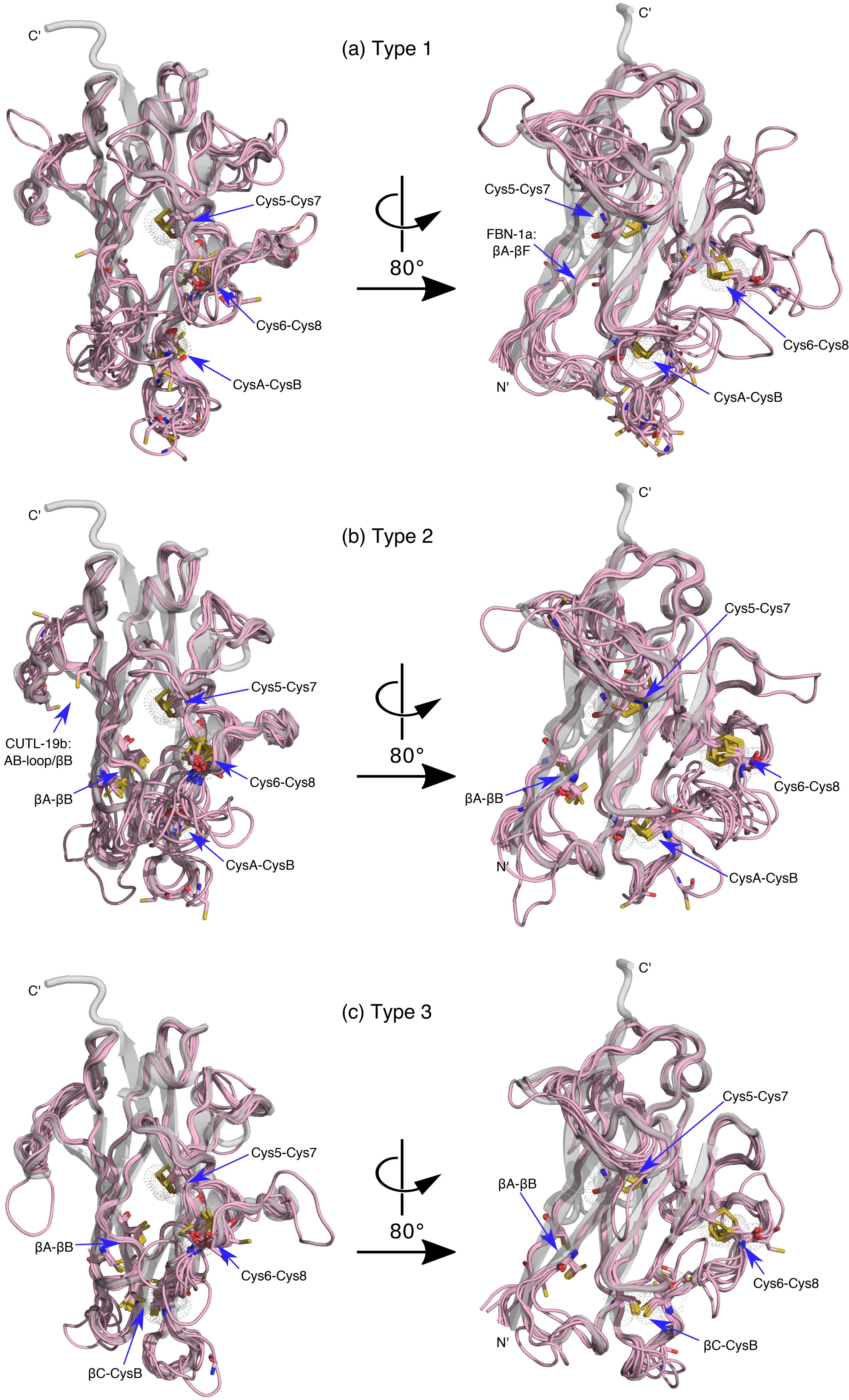

(a) Type 1
C'
Cys5-Cys7
Cys6-Cys8
CysA-CysB
80°
FBN-1a:
βA-βF
N'
(b) Type 2
CUTL-19b:
AB-loop/βB
βA-βB
(c) Type 3
βC-CysB
